# Exploring the Impact of the Biofloc Rearing System and an Oral WSSV Challenge on the Intestinal Bacteriome of *Litopenaeus vannamei*

**DOI:** 10.3390/microorganisms6030083

**Published:** 2018-08-08

**Authors:** Mariana R. Pilotto, André N. A. Goncalves, Felipe N. Vieira, Walter Q. Seifert, Evelyne Bachère, Rafael D. Rosa, Luciane M. Perazzolo

**Affiliations:** 1 Laboratory of Immunology Applied to Aquaculture, Department of Cell Biology, Embryology and Genetics, Federal University of Santa Catarina, 88040-900 Florianópolis, SC, Brazil; maryrp@gmail.com (M.R.P.); anicolau85@gmail.com (A.N.A.G.); rafael.d.rosa@ufsc.br (R.D.R.); 2 Laboratory of Marine Shrimp, Department of Aquaculture, Federal University of Santa Catarina, 88040-900 Florianópolis, SC, Brazil; felipe.vieira@ufsc.br (F.N.V.); walter.seiffert@ufsc.br (W.Q.S.); 3 Ifremer, UMR 5244, IHPE Interactions-Hosts-Pathogens-Environment, UPVD, CNRS, Université de Montpellier, 34095 Montpellier, France; ebachere@ifremer.fr

**Keywords:** biofloc technology (BFT), penaeid shrimp, gut microbiota, White spot syndrome virus, 16S rRNA gene sequencing

## Abstract

We provide a global overview of the intestinal bacteriome of *Litopenaeus vannamei* in two rearing systems and after an oral challenge by the White spot syndrome virus (WSSV). By using a high-throughput 16S rRNA gene sequencing technology, we identified and compared the composition and abundance of bacterial communities from the midgut of shrimp reared in the super-intensive biofloc technology (BFT) and clear seawater system (CWS). The predominant bacterial group belonged to the phylum Proteobacteria, followed by the phyla Bacteroidetes, Actinobacteria, and Firmicutes. Within Proteobacteria, the family Vibrionaceae, which includes opportunistic shrimp pathogens, was more abundant in CWS than in BFT-reared shrimp. Whereas the families Rhodobacteraceae and Enterobacteriaceae accounted for almost 20% of the bacterial communities of shrimp cultured in BFT, they corresponded to less than 3% in CWS-reared animals. Interestingly, the WSSV challenge dramatically changed the bacterial communities in terms of composition and abundance in comparison to its related unchallenged group. Proteobacteria remained the dominant phylum. Vibrionaceae was the most affected in BFT-reared shrimp (from 11.35 to 20.80%). By contrast, in CWS-reared animals the abundance of this family decreased from 68.23 to 23.38%. Our results provide new evidence on the influence of both abiotic and biotic factors on the gut bacteriome of aquatic species of commercial interest.

## 1. Introduction

Over the past twenty years, the shrimp industry has faced critical challenges related to environmental issues (scarce water quality) and infectious diseases caused by viruses and bacteria [1,2]. Among the infectious diseases of penaeid, the White spot syndrome (WSS) and the Acute hepatopancreatic necrosis disease (AHPND, previously referred as “Early mortality syndrome”) are currently the most serious threats to shrimp farmers. WSS is caused by the White spot syndrome virus (WSSV), whereas the AHPND is an emerging bacteriosis caused by virulent strains of *Vibrio parahaemolyticus* and *V. harveyi* [3,4,5]. Both infections have been causing global losses to the shrimp farming industry, which call for the efforts of both researchers and farmers toward the development of strategies to prevent disease outbreaks. In this context, the adoption of on-farm biosecurity practices has been a needful action to limit the pathogen entrance into the cultures [6].

Furthermore, traditional culture systems are responsible for generating an immense amount of excess organic pollutants, which requires continuous replacement of the pond water through exchange [7]. Among on-shrimp farm biosecurity practices, the BioFloc super-intensive Technology (BFT) has emerged as a promising alternative culture system. This system is based on the principle of waste nutrients recycling, where animals are stocked at high densities (minimum of 300 g of biomass per square meter) and little or no water exchange is performed [8,9]. Water exchange is a procedure to be avoided, once the influent water and shrimp larvae represent the most significant ways to enter pathogens in the farms [10]. Bioflocs are formed by a rich microbiota composed of fungi, protozoa, zooplankton, microalgae, and heterotrophic bacteria responsible for removing the excess ammoniacal nitrogen and maintaining stable levels of nutrients in the water [8,11]. Moreover, microbial flocs are also used as food for the animals and their consumption can induce an increase in shrimp growth performance [12,13] and robustness [14,15]. It is widely believed that BFT culture improves the crustacean immunity leading to high survival rates even under bacterial and viral infections [14,16]. Although the mechanisms underlying shrimp robustness are not yet understood, a continuous immunostimulation condition is rather expected considering the abundance of microbial-associated molecular patterns (MAMPs) present in BFT systems [14,15].

Since the aquatic environment can influence the microbiota composition and abundance [17], studies focusing the BFT contribution on the establishment of shrimp intestinal microbiota are highly required. In addition, it is now well established that commensal microbiota is essential for the correct functionality of the host physiology [18]. Surprisingly, the characterization of the microbial communities present in the digestive tract of cultured shrimp species has been only recently uncovered [19,20,21]. To our knowledge, only one report regarding the description of the intestinal bacterial communities of a penaeid species (*Litopenaeus stylirostris*) reared in the BFT system is available in the literature [22]. In addition, nothing is known about the bacteriome plasticity in shrimp infected by the WSSV, one of the most important pathogens to shrimp farming. In this context, we aimed to characterize the abundance and composition of the intestinal bacterial communities of the most important penaeid species, *Litopenaeus vannamei*, reared in BFT and a clear seawater system. Likewise, the plasticity of the midgut bacteriome from shrimp challenged by WSSV was investigated. Our results bring new evidence of the influence of the biofloc culture and the viral challenge on the shrimp bacteriome, providing new insights into future studies regarding the role of microbiota on the intestinal immunity of cultured penaeid.

## 2. Materials and Methods

### 2.1. Animals and Experimental Design

**Figure 1:**
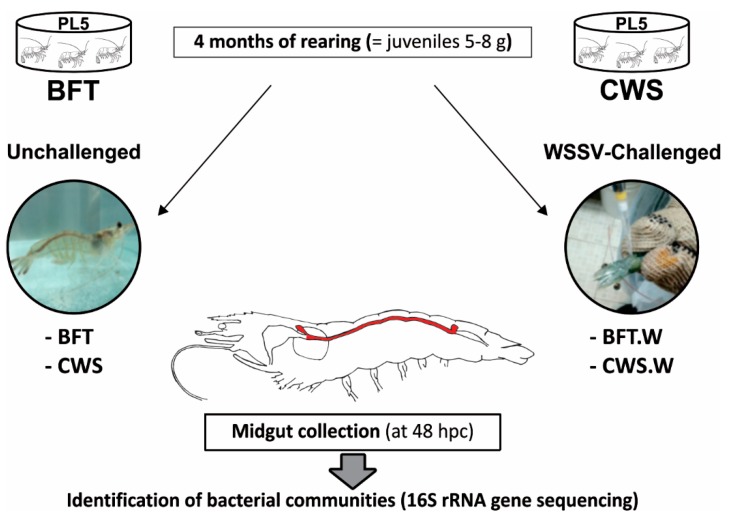
Post-larvae stage 5 (PL_5_ = five-day-old) from lineage HB12 of *Litopenaeus vannamei* were cultivated during four months in two culture systems: Biofloc Technology (BFT) (4 tanks) and clear seawater system (CWS) (4 tanks), at an initial stocking density of 300 and 20 PL_5_.m^−3^, respectively. White spot syndrome virus (WSSV)-free juvenile shrimp (5–8 g) from each condition (*n* = 80) were individually challenged with WSSV by the oral route (5 × 10^6^ genome viral copies). The remaining animals (*n* = 40/condition) were not handled. At 48 h post-challenge (hpc), midguts from unchallenged (BFT and CWS) and WSSV-challenged (BFT.W and CWS.W) shrimp (*n* = 40/condition) were collected and processed for 16S RNA gene sequencing.

Post-larvae stage 5 (PL_5_ = five-day-old) from lineage HB12 of *Litopenaeus vannamei* were obtained from Aquatec LTDA (Canguaratema, Rio Grande do Norte, Brazil) and transferred to the Laboratory of Marine Shrimp (Federal University of Santa Catarina, Florianópolis, Brazil). The biofloc culture was initially established in a 50 m^3^ matrix tank according to [23]. The experimental design is shown in Figure 1. PL_5_ was randomly allocated into 8 indoor tanks of 9 m^3^, according to the following types of growing conditions: BioFloc Technology system (BFT; 4 tanks) and clear seawater system (CWS; 4 tanks), at an initial stocking density of 300 and 20 PL_5_ m^−3^, respectively. The tanks were continuously aerated (dissolved oxygen > 5 mg L^−1^) and kept under controlled temperature (29 ± 1°C) and salinity (34–35). Post-larvae were fed four times a day with the commercial Guabi Potimar feed. The daily seawater exchange rate of the CWS tanks was 80%, whereas in the BFT tanks the evaporated water was replaced with freshwater. The amount of solids present in BFT units was usually maintained between 400 and 600 mg L^−1^ and when in excess, solids were removed by using settling tanks (100 L) [23]. The water’s physicochemical parameters were analyzed according to [23]. After four months, when the PL_5_ reached the juvenile stage (5–8 g), around 30% of the animals from each tank/group were randomly selected to confirm that the shrimp were free of WSSV by using the Nested-PCR assay described by [24]. Finally, shrimp (*n* = 120/culture system) were transferred to the Laboratory of Immunology Applied to Aquaculture (Federal University of Santa Catarina, Florianópolis, Brazil).

### 2.2. WSSV Per Os Challenge and Midgut Collection

The viral inoculum was prepared as previously described in [25], with some modifications. In brief, the abdomen tissues of WSSV-infected shrimp were homogenized in a Tris-saline solution (20 mM Tris, 330 mM NaCl, pH 7.4) (1:10 *w/v*) using a hand blender. The tissue homogenate was centrifuged twice (2000× *g* for 20 min and 9000× *g* for 10 min) at 4 °C and the supernatant filtered (0.45 µm), aliquoted and stored at −80 °C [25]. Eighty shrimp from each condition were challenged with WSSV inoculum (100 µL containing 5 × 10^6^ viral copies) that was gently flowed into the oral cavity by using a micropipette. No mortalities were recorded. After 48 h post-challenge, midguts from unchallenged (BFT and CWS) and challenged (BFT.W and CWS.W) shrimp were aseptically dissected, washed twice (70% ethanol and sterile seawater), and stored in 70% ethanol for further DNA extraction and metagenomic sequencing. The WSSV infection was confirmed by Nested-PCR [24]. This time point (48 h) was chosen for midgut collection due previous results with *L. vannamei* infected with WSSV which start to die from 72 h post-challenge [25,26].

### 2.3. Genomic DNA (gDNA) Extraction

For total genomic DNA (gDNA) extraction, individual midguts stored in 70% ethanol were rinsed in the Tris-saline solution and homogenized with a lysis solution containing 0.1 M Tris-HCl (pH 8.5), 0.1 M NaCl, 0.05 M EDTA (pH 8.0), 1% SDS and proteinase K (Sigma-Aldrich, St. Louis, MO, USA) (final concentration 0.25 µg µL^−1^). After incubation for 1 h at 55°C, 3 M potassium acetate (1:2; *v/v*) was added to the mix, following incubation for 30 min at 4°C and centrifugation at 14,000× *g* for 10 min. gDNA precipitation was performed using isopropanol (1:2; *v/v*) and the mix was immediately centrifuged at 14,000× *g* for 30 min at 4 °C. The gDNA pellet was washed (70% ethanol), resuspended in ultrapure water and the gDNA concentration and purity were verified by using the NanoVue plus™ spectrophotometer. Individual gDNA samples were pooled to compose four 16S rRNA libraries: BFT, CWS, BFT.W, and CWS.W. The same concentration of template DNA from each pooled sample (100 ng) was used for the following amplification methods.

### 2.4. 16S rRNA Gene Library Preparation and High Throughput Sequencing

Firstly, a PCR amplification was performed using a primer pair based on the V3-V4 hypervariable region of the prokaryotic 16S rRNA gene in the midgut samples of *L. vannamei*: 341 Fw (5′-CCT AYG GGR BGC ASC AG-3′) and 806 Rv (5′-GGA CTA CNN GGG TAT CTA AT-3′) [27]. The PCR reaction was carried out in 50 µL containing 1 µL gDNA template (100 ng), 0.5 µM of each primer, 1× Phusion High-Fidelity PCR Master Mix [20 units mL^−1^ Phusion DNA polymerase, 0.2 mM each dNTP, 25 mM TAPS-HCl (pH 9.3), 50 mM KCl, 2 mM MgCl_2_, 1 mM β-mercaptoethanol and 400 µg mL^−1^ activated Calf Thymus DNA] (New England Biolabs, Ipswich, MA, USA). The PCR conditions were: initial denaturation at 98 °C for 30 s, 35 cycles of 98 °C for 5 s, 56 °C for 20 s, and 72 °C for 20 s, and a final extension step of 72 °C for 5 min. The quality of PCR products was verified by electrophoresis in 2% agarose gel. PCR product bands were purified by using Qiagen Gel Extraction Kit (Qiagen, Hilden, Germany) and libraries from each condition were sequenced by Illumina HiSeq 2500 Platform (GenOne Biotechnologies, Rio de Janeiro, Brazil).

### 2.5. Sequence Data Analysis

All Illumina sequencing data were filtered and analyzed using the QIIME 1.7.0 software package (Quantitative Insights Into Microbial Ecology) [28]. Firstly, the data were filtered to remove forward/reverse primers and barcode sequences considering quality phred ≥ Q20. Quality filtering on the data were performed under specific conditions to obtain the high-quality clean sequences [29]. Chimera sequences were removed using the UCHIME Algorithm (http://www.drive5.com/usearch/manual/uchime_algo.html) [30]. Operational Taxonomic Units (OTUs) were assigned (97% similarity) using the pick_closed_reference_otus.py scripts with the UCLUST method and annotated in the Greengenes 13_8 database reference (http://greengenes.lbl.gov/cgi-bin/nph-index.cgi) [31]. The similarity level used by GreenGenes assignment was ≥ 97%. Different indexes were selected to identify species richness (Chao1 [32] and ACE [33] estimators) and species diversity (Shannon index [34] and Simpson index [35]). Rarefaction curves of the expected species richness (using Chao1) with 95% confidence intervals were generated based on the cumulative sampling using make_rarefaction_plots.py script from QIIME software. Finally, Good’s coverage was used to estimate the sequencing depth. OTUs abundance information was normalized using a number of standard sequences corresponding to the biggest sample. Subsequent analysis of alpha and beta bacterial diversities were performed based on OTUs normalized data using QIIME software. To estimate the beta diversity (differences between samples), a principal coordinates analysis (PCoA-Weighted UniFrac distance analysis) was conducted with the software package of Fathom Toolbox for Matlab (http://www.marine.usf.edu/user/djones). Venn diagram and relative abundance graphs were performed using the R script (http://www.r-project.org/) and the ggplot2 software (ggplot2.org).

## 3. Results and Discussion

### 3.1. Overview of the Illumina Sequencing, Diversity, and Richness of Bacteria

We have explored here the bacterial communities’ dynamics (abundance and phylogenetic composition) in the shrimp midgut in response to two important abiotic and biotic factors related to shrimp farming (culture system and viral infection) by assessing 16S rRNA gene sequencing. The BFT water parameters are shown in Table S1. Two bacterial 16S rRNA gene libraries have been generated from the midguts of shrimp cultured in a BioFloc Technology (library “BFT”) and in a Clear Seawater System (library “CWS”). Likewise, two other libraries were generated from the midguts of shrimp challenged with the WSSV using a *per os* method (libraries “BFT.W” and “CWS.W”). We focused on investigating the midgut portion from the shrimp gut because it lacks the chitinous layer of the cuticle normally found in the other portions of the intestine (foregut and hindgut). Moreover, shrimp midgut possesses an epithelial layer that is protected by a semi-permeable, non-permanent peritrophic matrix [36,37], thereby representing a potential route of pathogen entry [38].

**Figure 2:**
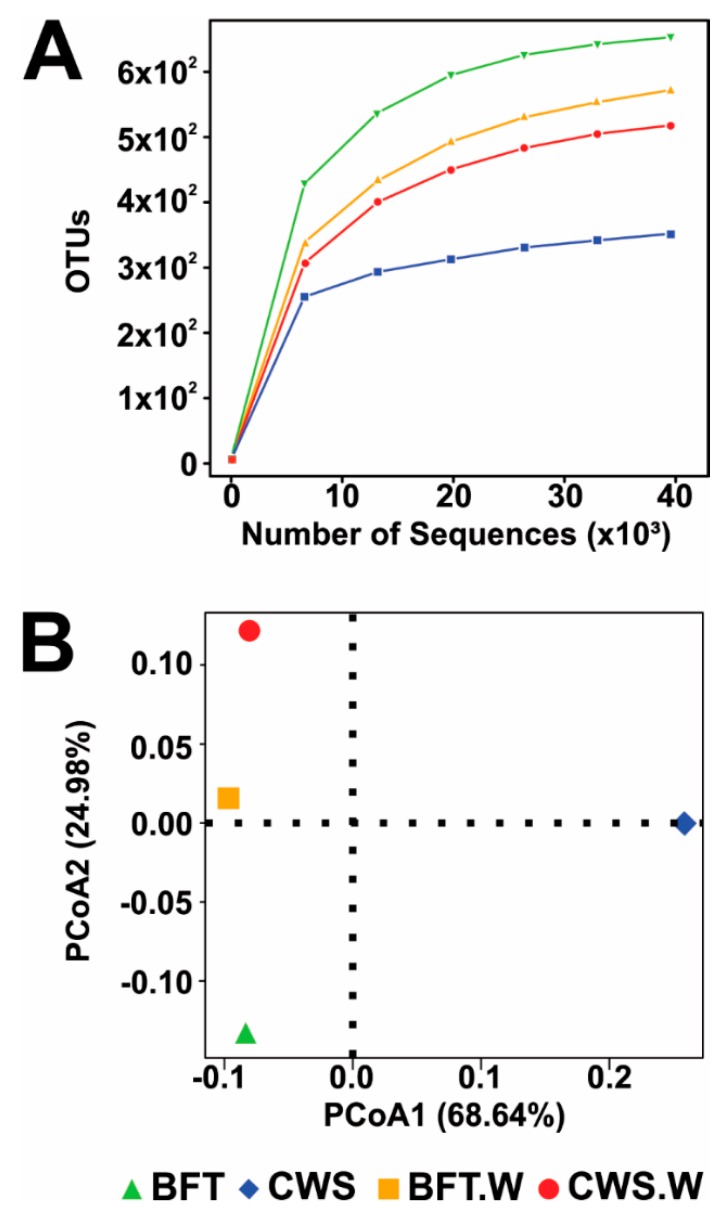
Sequencing coverage analysis by rarefaction curve (**A**) and Principle coordinate analysis (**B**) indicating the similarities and dissimilarities between bacterial communities present in the midgut of *Litopenaeus vannamei* reared in two different rearing systems (BFT: biofloc; CWS: clear seawater) and after an oral challenge by the White spot syndrome virus (BFT.W: shrimp reared in BFT and challenged by the WSSV; CWS.W: shrimp reared in CWS and challenged by the WSSV).

The full dataset for this project has been deposited at NCBI Sequence Read Archive (SRA accession: SRS3345544, SRS3345545, SRS3345546, SRS3345547). A total of 275,561 raw sequences were generated using the Illumina Hi-Seq 2500 platform sequencing and, after quality trimming, 185,745 high-quality sequences were used for further analysis (Table S2). From those high-quality sequences, 39,938 were generated from BFT, 47,030 from CWS, 50,836 from BFT.W and 47,941 from CWS.W. Sequences were clustered into 1299 Operational Taxonomic Units (OTUs) at 97% sequence similarity using the Greengenes 13_8 reference OTU collection database as reference. Analysis of the rarefaction curves from the 16S rRNA gene sequences of all the samples reached a plateau (saturation), indicating that the sequencing depth was sufficient to cover the bacterial communities of the *L. vannamei* midgut (Figure 2A), confirmed by the Good’s coverage analysis (Table 1). Estimation of phylotype richness was calculated according to the bias-corrected Chao1 estimator and the abundance-based coverage estimator (ACE). Chao1 scores ranged from 487.07 to 674.05, while ACE predicted a range from 408.01 to 672.01 phylotypes (Table 1), indicating that shrimp samples from BFT exhibited a higher phylotype richness than the CWS samples.

In order to compare the bacterial communities’ diversity from each sample, Shannon and Simpson indices were calculated from the obtained OTUs. Shannon indexes were 6.24 for BFT, 4.13 for CWS, 5.79 for BFT.W, and 4.55 for CWS.W (Table 1). Simpson indexes of diversity were 0.96 for both BFT and BFT.W, 0.79 for CWS and 0.89 for CWS.S. High Simpson indexes in BFT-reared animals indicate a higher bacterial communities’ diversity in the midguts from shrimp reared in BFT than in CWS. Indeed, BFT represents a rich microbial environment that provides the establishment of many different bacterial communities in the shrimp gut [22] and, the composition of the *L. vannamei* midgut seems to be closely related to the rearing system. The Principal coordinate analysis (PCoA) plot data revealed the similarities and dissimilarities between bacterial communities present in the midgut of *L. vannamei* reared in different systems (BFT or CWS) and after the WSSV challenge (BFT.W and CWS.W). While BFT, BFT.W and CWS.W were grouped in a same clade, CWS formed a separated group (Figure 2B). These findings indicate thorough differences in the intestinal bacterial composition of shrimp reared in CWS when compared to BFT-reared animals or facing a viral challenge (BFT.W and CWS.W).

**Figure 3:**
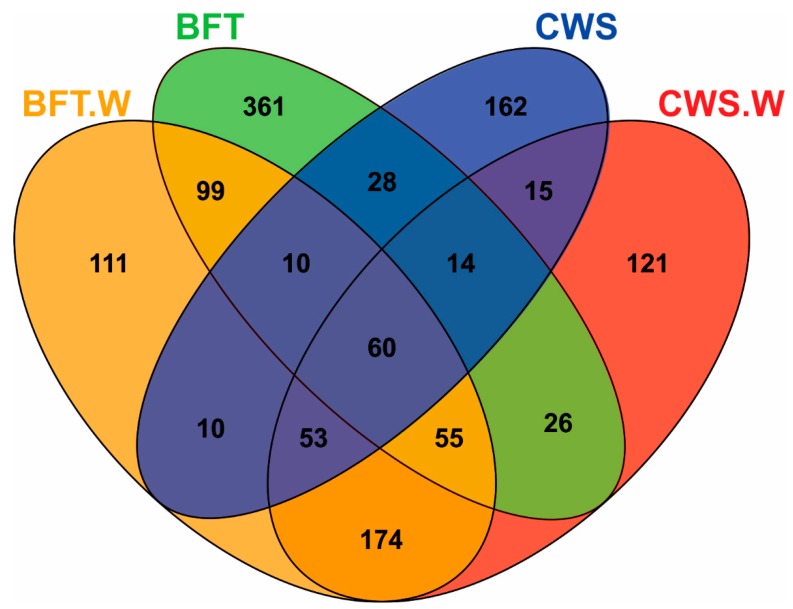
Venn diagram showing unique and shared operational taxonomic units (OTUs) of midgut bacteria of *Litopenaeus vannamei* reared in biofloc (BFT) and clear seawater (CWS) and challenged by the WSSV (BFT.W and CWS.W), by using a *per os* method.

Venn diagram analyses revealed significant differences in the frequency distribution of bacterial OTUs according to the culture system (BFT and CWS) and viral challenge (Figure 3). Midguts of animals reared in BFT exhibited a larger number of OTUs when compared with those from CWS. 571 OTUs were exclusively found in shrimp reared in bioflocs (361 in BFT, 111 in BFT.W and 99 in both groups), whereas 298 OTUs were exclusive from shrimp reared in clear seawater (162 in CWS, 121 in CWS.W and 15 in both conditions). The higher amount of exclusive OTUs from the BFT samples could reflect the diversity microbioma of the bioflocs environment. The viral challenge leads to the appearance of exclusive OTUs in each rearing condition: 111 OTUs for BFT.W and 121 for CWS.W (Figure 3**)**. Furthermore, exclusive OTUs (*n* = 174) were shared only by the challenged animals of both rearing condition. These findings suggest that this bacterial community displacement in the midgut is related to the virus presence. In the human gut, the influenza virus promotes the depletion of some bacteria communities and the enrichment of others, leading to an imbalance of the microenvironment condition, a state known as dysbiosis [39]. Studies on microbiota shrimp-virus interaction related to the BFT rearing system deserve to be investigated in the future. Finally, 60 OTUs were shared among all samples, which represented 4.61% of the total OTUs. This bacteria subset present in all groups could represent relevant microorganisms to the fundamental structure and function of the shrimp intestinal microbiota [40]. The list of shared and unique OTUs annotation indicated in the Venn diagram is provided in Supplemental Table S3.

### 3.2. Influence of Rearing Conditions on the Bacterial Communities of Shrimp Midgut

**Figure 4:**
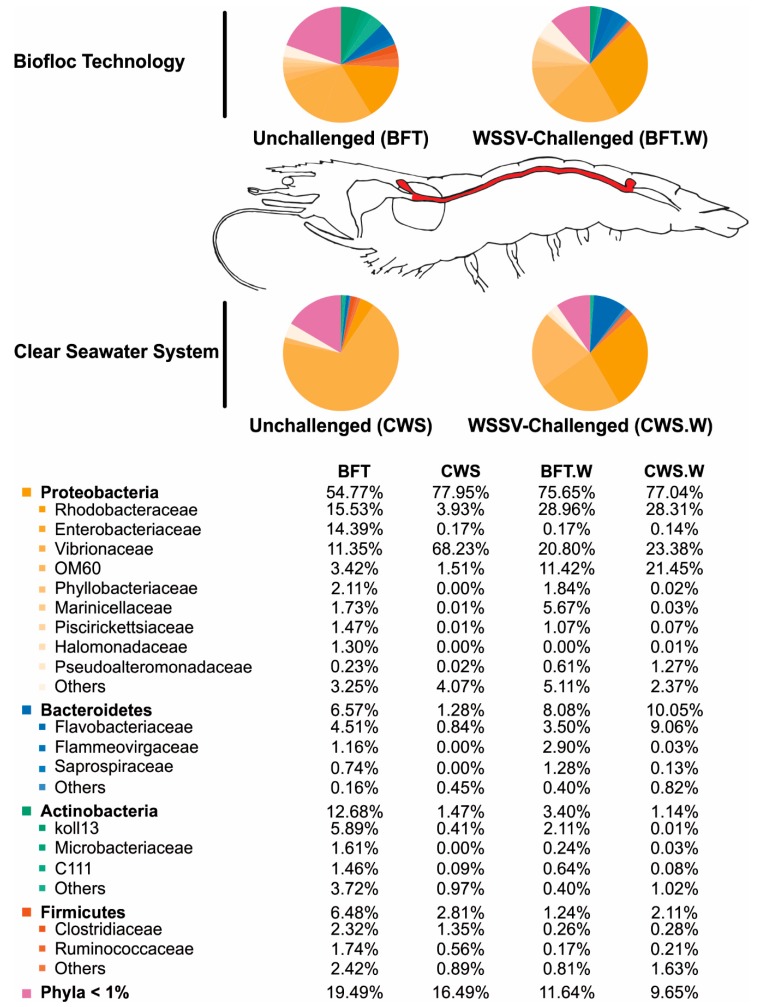
Relative abundance of the most prevalent bacterial phyla and families identified in the midgut of *Litopenaeus vannamei* (highlighted in red in the not-to-scale image) reared in biofloc (BFT) and clear seawater (CWS), and at 48 h after an oral challenge by the White spot syndrome virus (BFT.W and CWS.W).

It is widely believed that the commensal microbiota composition in adult arthropods appears to be intimately related to the initial exposure to microorganisms on precocious stages of their life [41,42]. Based on that, we performed the shrimp grown in BFT and CWS for four months, from post-larvae aged for 5 days (PL_5_) until the juvenile stage (5–8 g) (Figure 1). In our analysis, the obtained high-quality 16S rRNA gene sequences were classified into 33 prokaryotic phyla that belong to the domain Bacteria. The most representative phyla identified in the *L. vannamei* midgut were Proteobacteria, Bacteroidetes, Actinobacteria, and Firmicutes. However, the frequency distribution of the intestinal bacterial communities differed according to the rearing system (Figure 4).

For both rearing conditions, Proteobacteria was the most abundant phylum associated with the midgut of *L. vannamei*, which is consistent with previous studies using the same penaeid species [43,44,45,46]. The relative abundance of Proteobacteria was higher (77.95%) in CWS than in BFT (54.77%). Within this phylum, Gammaproteobacteria was the most dominant class in the midgut of shrimp reared in both conditions, despite the fact that in CWS the relative abundance was superior (73%) to those of BFT (39%) (data not shown). Interestingly, this class comprises several medically and ecologically important groups of Gram-negative bacteria, including several pathogens to human and animals that belong to the Enterobacteriaceae, Vibrionaceae, and Pseudomonadaceae families. Particularly, Vibrionaceae accounted for 68.23% from the total bacteria present in the midgut of whiteleg shrimp cultivated in CWS, in contrast with 11.35% of the intestinal bacteriome of shrimp cultivated in BFT (Figure 4). In a recent study performed with the Western blue shrimp, *L. stylirostris*, the intestinal microbiota was characterized in animals reared in BFT and CWS, as well the bacterial communities from the water rearing system [22]. Vibrionaceae was less abundant into BFT rearing water (0.1%) than in the CWS water (4.7%), whereas Vibrionaceae communities had a very similar abundance in the Western blue shrimp intestine reared in both culture systems (54.5% and 57.6% to BFT and CWS, respectively). In addition, it has been seen that the BFT altered the species composition of the *Vibrio* community in *L. vannamei* hepatopancreas compared to shrimp reared in CWS, and shrimp showed better health status than those cultivated in CWS [47].

The genus *Vibrio* is composed by fast-growing aquatic Gram-negative bacteria, able to colonize the digestive tract of different animals, including penaeids [48]. However, many *Vibrio* species are considered opportunistic pathogens for shrimp under stressful conditions, such as poor nutrition, low water quality, and immune depression [48]. In cultured shrimp, this bacterial group has been repeatedly implicated in gastro-intestinal diseases, leading to high mortality in shrimp farming worldwide [49,50]. For instance, a new emerging vibriosis named acute hepatopancreatic necrosis disease (AHPND) that is caused by virulent strains of *V. parahaemolyticus* and *V. harveyi* has seriously impacted the shrimp industry worldwide since its first description in China, in 2009, causing up to 100% mortality in a few days of rearing [4,5]. The indiscriminate use of antibiotics attempting to protect the farms from bacteriosis has led to the spread of multiple resistant strains of *Vibrio* [51]. As an alternative to the indiscriminate use of antibiotics in the shrimp farms, probiotics and bioflocs are current environmentally friendly technologies based on *in situ* microorganism production and aiming the control of infectious diseases [8,48]. In this context, the BFT system arises as a “natural probiotic” culture strategy [52]. Microorganisms present in BFT water could act against pathogenic bacteria by competition for substrate and nutrients, producing inhibitory compounds, and interfering in the bacterial *quorum*-sensing communication [8,9,16,47,53].

Interestingly, two Proteobacteria families, Rhodobacteraceae and Enterobacteriaceae, were more abundant in the midgut of shrimp reared in BFT than in CWS, representing around 15% in BFT and less than 4% in CWS (Figure 4). Rhodobacteraceae were also more abundant in the intestine of Western blue shrimp reared in the BFT system than in CWS [22]. This bacterial family is commonly found associated with biofilms and other aquatic surfaces, usually being the dominant and the first bacterial group to colonize these places [54]. The role of this bacterial family in the shrimp intestinal microbiota is not well understood. Nonetheless, it is believed that the BFT system can favor the presence of this bacteria family due to its high concentration of suspended solid [55] that can be used as growth sites by Rhodobacteraceae [22]. Interestingly, Rhodobacteraceae members can establish an antagonistic activity limiting the survival of pathogenic *Vibrio* [56]. Therefore, we could hypothesize that the higher abundance of Rhodobacteraceae in the midgut of *L. vannamei* reared in BFT could be associated with a lower abundance of Vibrionaceae.

The other predominant phyla in the midgut of whiteleg shrimp reared in BFT when compared with CWS were Bacteroidetes, Actinobacteria, and Firmicutes (Figure 4). Bacteroidetes is a dominant member of marine heterotrophic bacterioplankton and it is frequently found colonizing macroscopic organic matter particles [57]. Flavobacteriaceae represents one of the most abundant microorganisms in aquatic environments [58] and in our study, Flavobacteriaceae was the main Bacteroidetes family present in the midgut of *L. vannamei* reared in BFT (4.51%) and CWS (0.84%). Finally, the last two phyla composing the intestinal bacteriome of *L. vannamei* were Actinobacteria and Firmicutes. Actinobacteria corresponded to 12.68% in BFT and 1.47% in CWS, whereas the relative abundance of Firmicutes was 6.48% in BFT and 2.81% in CWS. The lower abundance of both phyla in the intestinal microbiota of *L. vannamei* has been recently documented [43,44]. Finally, two bacterial species affiliated with Firmicutes group, *Bacillus* and *Lactobacillus*, with potential use as probiotics in shrimp aquaculture [59] were poorly represented in our study (Bacilli class represented 1% in both rearing conditions).

Our findings indicate that the bacteriome of shrimp reared in BFT was more diversified and rich when compared to that from animals reared in clear seawater, where the predominant bacterial community was Vibrionaceae. The water from BFT is especially rich in organic matter and suspended particles, which can favor bacteria that use organic matter and nitrogen compounds for growth [55,60]. In addition, the BFT rearing system apparently causes important modifications in shrimp midgut microbiota compared to CWS, which corroborates the fact that the microbiota from the digestive tract of the aquatic animals is directly influenced by the environment [22,59,61,62]. In addition, the idea of considering the BFT as a “natural probiotic system” [52] has important consequences to the intestinal microbiota. The BFT could act internally and/or externally to the shrimp body, an effect promoted by large groups of microorganisms, but mainly bacteria [52].

Regarding the beneficial effects of the BFT system, it has also been seen that bioflocs can act as immunostimulants, enhancing the shrimp innate immune system [14,63], even altering the expression of genes related to the shrimp immune response [15], which could be attributed to the ability of the BFT to induce changes in shrimp microbiota. The host immune system and its microbiota are considered to have a close relationship. The microbiota is required for intestinal immune maturation and, on the other hand, the host’s immune responses regulate the structure and composition of its intestinal microbiota [64]. The host’s immune responses are dependent on sophisticated systems for recognizing and also differentiating beneficial and pathogenic microorganisms [65], and the direct contact with the environment makes this recognition even more important for aquatic animals [66]. In shrimp, the complexity of the interface between the host’s immune system and its gut microbiota is still unknown. However, the RNAi-mediated knockdown of the ALF*Pm*3 gene (a member of the ALF antimicrobial peptide family) resulted in the rapid death of the animals, which was attributed to an uncontrolled bacterial growth [67]. Overall, these results show that gut microbiota of shrimp is altered by the rearing environment. The midguts of BFT-reared shrimp were composed mainly by bacteria that uses organic matter to grow on. As it has been previously found that the BFT system could offer multiple benefits to shrimp, we can now investigate the correlation between these beneficial consequences to the change in the gut microbiota.

### 3.3. Shrimp Intestinal Microbiota Plasticity in Response to a Viral Challenge

The most impacting results of our study were to characterize the bacterial communities shift in the shrimp midgut challenged by WSSV. To the best of our knowledge, there is only one study on the shift of gut microbiota in response to the WSSV infection evaluated in the Chinese mitten crab *Eriocheir sinensis* [68]. This is the first study investigating the effect of a viral pathogen on the intestinal microbiota of a penaeid species reared in the BFT system. To achieve this purpose, we chose the oral route of infection (Figure 1), considering the common cannibalism practice among shrimp. Although shrimp midguts were all positive for the WSSV (Nested-PCR approach), no mortalities were recorded at 48 h post-challenge. *In silico* analysis showed a displacement in the bacterial communities present in the midgut of shrimp challenged with the WSSV.

In our study, the bacterial composition of *L. vannamei* midgut was affected by the presence of a viral pathogen, similarly to that observed in previous studies using WSSV [68] and *Vibrio* [19,44,59]. Proteobacteria remained the most predominant phylum in the *L. vannamei* midgut even after the viral challenge (Figure 4). Similar results were shown in *L. vannamei* after bacterial infections caused by *V. parahaemolyticus* [19] and *V. harveyi* [44]. The abundance of Proteobacteria in the midgut of *L. vannamei* reared in BFT increased after WSSV challenge, from 54.77% (BFT) to 75.65% (BFT.W). However, in shrimp reared in CWS, its abundance was not affected (77.95% for CWS and 77.04% for CWS.W). Within Proteobacteria, Vibrionaceae was the most affected bacterial family by the presence of the virus. In BFT-reared shrimp, the abundance of Vibrionaceae doubled after WSSV challenge, from 11.35 to 20.80% (Figure 4). In contrast to the results obtained in BFT rearing, the relative abundance of the Vibrionaceae in the midgut of *L. vannamei* reared in CWS decreased drastically after WSSV challenge, from 68.23% (CWS) to 23.38% (CWS.W). The WSSV challenge also increased the abundance of Vibrionaceae in the gut of mitten crab *Eriocheir sinensis* [68]. As suggested by [68], the virus could deplete the host’s immune system, leading to the increase of opportunistic pathogens, such as bacteria from the *Vibrio* genus. In our study, the Rhodobacteraceae family increased in both rearing conditions after the WSSV challenge (Figure 4). The highest increase was found in CWS-reared shrimp, from 3.93 to 28.31%. This increase could be due to the Vibrionaceae family decrease in the same condition, once Rhodobacteraceae members can have an antagonistic effect upon pathogenic bacteria from the Vibrionaceae family [56]. Surprisingly, the Vibrionaceae abundance increased in the midgut of BFT-reared shrimp from 11.35 to 20.80%, after the WSSV challenge. As previously described, this family is composed not only by bacteria commonly found inhabiting shrimp midgut, but also by opportunistic pathogens which, under favorable circumstances, could lead to severe shrimp diseases [48].

Regarding the Bacteroidetes phylum, the WSSV presence also led to an intensification in the abundance of this bacteria in shrimp reared in both conditions, although this increase was higher once again in CWS-reared animals than in BFT (from 1.28 to 10.05% in CWS, and from 6.57 to 8.98% in BFT). An increase in the Bacteroidetes family was also found in the midgut of the *L. vannamei* infected by *V. parahaemolyticus* [19] and in shrimp diseased by an unknown etiological agent [59]. The abundance of the third most representative phylum, Actinobacteria, decreased in the midgut of BFT-reared shrimp (from 12.68 to 3.40% in BFT.W), but was unchanged in animals cultured in CWS (1.47% and 1.14% in CWS and CWS.W, respectively). Similar results were found in *L. vannamei* after *V. parahaemolyticus* [19] and *V. harveyi* [44] infections and in shrimp diseased by an unknown etiological agent [59]. In our study, the Firmicutes phylum also decreased in *L. vannamei* midgut after WSSV challenge from 6.48% in BFT to 1.24% in BFT.W and, as observed for Actinobacteria, no changes were observed in CWS-reared shrimp (2.81% and 2.11% in CWS and CWS.W, respectively). Similarly to our findings, [68] also found a decrease in the abundance of Firmicutes in the digestive tract of the Chinese mitten crab at 48 h after WSSV infection. In *L. vannamei*, WSSV challenge apparently led to a more homogeneous distribution of bacterial population composition, as Rhodobacteraceae, Enterobacteraceae, and Vibrionaceae, in the midgut of shrimp reared in both culture systems, BFT, and clear seawater (Figure 4). Interestingly, these same bacterial families exhibited abundance quite differently than before the viral challenge.

The presence of a pathogen, such as a virus, can provoke a displacement in the microbial communities leading to a population imbalance or dysbiosis [64,69]. When the host fails to appropriately regulate the gastrointestinal microbiota through the immune system, the host can become increasingly susceptible to opportunistic infections [70]. Microbial dysbiosis might profoundly impact the development and physiological function of their hosts [71,72]. However, the potential displacement in the midgut bacteriome of *L. vannamei* challenged with WSSV could have been temporary. Herein, we evaluated only one-time point (48 h) after the viral challenge. In another study, the bacterial communities composition in the *L. vannamei* midgut was regained at 72 h after *V. harveyi* challenge, but not at 48 h post-challenge where it was quite modified [44]. In contrast, BFT-reared penaeid and *Artemia franciscana* showed high survival rates in the presence of *V. parahaemolyticus*, the etiological agent of AHPND [16,73]. In addition, components from the bioflocs system can inhibit in vitro the *V. harveyi* growth [16] and induce in vivo the modulation of the shrimp intestinal microbiota [61]. It appears that the disruption of the bacterial *quorum*-sensing is the cause of the protective action of bioflocs [16], but the precise mode of action and the interference of the bioflocs against pathogens need further research.

## 4. Conclusions

By using a high-throughput sequencing technology, we have characterized the intestinal bacteriome of the most important cultivated shrimp species, *L. vannamei*, and assessed the influence of the BFT rearing and of the WSSV challenge on the composition and abundance of the bacterial communities. The bacterial composition from the midgut of shrimp reared in bioflocs was more rich and diverse than that from clear seawater. The predominant bacterial group belonged to the phylum Proteobacteria (Rhodobacteraceae, Enterobacteriaceae and Vibrionacea), followed by the phyla Bacteroidetes (Flavobacteriaceae), Actinobacteria and Firmicutes. Vibrionaceae was more abundant in the CWS group than in BFT-reared shrimp (68.23% and 11.35% from total bacterial communities, respectively). The bacterial composition of *L. vannamei* midgut was affected by the WSSV challenge. Vibrionaceae was the most affected bacterial family and its abundance doubled in the midgut of BFT-reared shrimp after viral challenge, while in CWS-reared shrimp decreased drastically. In addition, the WSSV challenge apparently led to a more homogeneous distribution of bacterial population composition, as Rhodobacteraceae, Enterobacteraceae, and Vibrionaceae, in the midgut of shrimp reared in both culture systems, BFT and CWS. The changes in the gut bacteria diversity associated to the WSSV challenge could indicate a displacement in the intestinal microbial communities leading to the dysbiosis condition. Knowing the intestinal bacterial populations of shrimp reared in BFT and during WSSV infection is a relevant step to understanding the role of intestinal bacteriome microbiota on crustacean immune defenses against viral diseases. Although the molecular mechanisms involved in the control and regulation of the shrimp gut microbiota is still largely unknown, the environmental conditions and the presence of infectious agents proved to be decisive factors influencing both the diversity and abundance of the bacterial communities. With these 16S rRNA sequencing data in hand, and given that penaeid shrimp is an excellent model for functional genomic studies, we can now investigate the shrimp effectors involved in host-microbiota interactions, but also the role of the commensal microbiota in the regulation of the shrimp gut immunity.

## Figures and Tables

**Table 1:** Bacterial communities’ diversity and richness in the midgut of *Litopenaeus vannamei* reared in Biofloc technology (BFT) or clear seawater system (CWS) and after a viral challenge.

Groups	OTUs	H’	1-D	Chao1	ACE	Good’s Coverage
BFT	653	6.24	0.96	674.05	672.40	0.99
CWS	352	4.13	0.79	487.07	408.01	0.99
BFT.W	572	5.79	0.96	624.99	627.61	0.99
CWS.W	518	4.55	0.89	542.84	552.32	0.99

OTUs: operational taxonomic units; H’: Shannon index; 1-D: Simpson index; ACE: abundance-based coverage estimator. BFT.W: shrimp reared in BFT and challenged by the White spot syndrome virus (WSSV); CWS.W: shrimp reared in CWS and challenged by the WSSV.

## Supplementary Materials

The following are available online at http://www.mdpi.com/2076-2607/6/3/83/s1, Table S1: Physicochemical parameters of the BFT water after four months of *Litopenaeus vannamei* rearing, Table S2: Overview of 16S-based Illumina Hi-Seq 2500 sequencing of *Litopenaeus vannamei* midgut reared in bioflocs (BFT) and clear seawater (CWS) and challenged with WSSV (BFT.W and CWS.W), Table S3: Shared and unique OTUs annotation from *Litopenaeus vannamei* midgut reared in bioflocs (BFT) and clear seawater (CWS) and challenged with WSSV (BFT.W and CWS.W), and the abundance of unique OTUs annotation from major bacterial phyla.
