# Supplementary material for: Exploring the Impact of the Biofloc Rearing System and an Oral WSSV Challenge on the Intestinal Bacteriome of Litopenaeus vannamei
Source: Microorganisms. 2018 Aug 8;6(3):83. doi: 10.3390/microorganisms6030083 (PMC6164277; doi:10.3390/microorganisms6030083)
Supplement: Supplementary file 1 [file microorganisms-06-00083-s001.zip › Table S3.pdf]

		Abundance of unique OTUs from major phyla (%)			
	**Number of unique OTUs**	**Proteobacteria**	**Bacteroidetes**	**Actinobacteria**	**Firmicutes**
**BFT**	362	17.1	3.6	8.8	37.0
**CWS**	162	34.0	8.6	6.8	9.3
**BFT.W**	111	31.5	9.0	1.8	17.1
**CWS.W**	121	26.4	10.7	5.8	14.9
**BFT.W x CWS.W**	174	27.6	15.5	0.6	32.2
**Shared**	60	46.6	15.0	6.6	6.6

BFT							
**OTUs**	**Kingdom**	**Phylum**	**Class**	**Order**	**Family**	**Genus**	**Specie**
OTU_304	Bacteria	Proteobacteria	Alphaproteobacteria	Caulobacterales	Caulobacteraceae	Brevundimonas	diminuta
OTU_1163	Bacteria	Proteobacteria	Alphaproteobacteria	Caulobacterales	Caulobacteraceae	Caulobacter	
OTU_1064	Bacteria	Proteobacteria	Alphaproteobacteria	RF32			
OTU_548	Bacteria	Proteobacteria	Alphaproteobacteria	Rhizobiales	Bradyrhizobiaceae	Bradyrhizobium	
OTU_1025	Bacteria	Proteobacteria	Alphaproteobacteria	Rhizobiales	Hyphomicrobiaceae	Devosia	
OTU_226	Bacteria	Proteobacteria	Alphaproteobacteria	Rhizobiales	Methylobacteriaceae		
OTU_1173	Bacteria	Proteobacteria	Alphaproteobacteria	Rhizobiales			
OTU_458	Bacteria	Proteobacteria	Alphaproteobacteria	Rhodobacterales	Rhodobacteraceae	Phaeobacter	
OTU_1085	Bacteria	Proteobacteria	Alphaproteobacteria	Rhodospirillales	Acetobacteraceae	Swaminathania	
OTU_921	Bacteria	Proteobacteria	Alphaproteobacteria	Rickettsiales			
OTU_1017	Bacteria	Proteobacteria	Alphaproteobacteria	Rickettsiales			
OTU_1009	Bacteria	Proteobacteria	Alphaproteobacteria	Rickettsiales			
OTU_338	Bacteria	Proteobacteria	Alphaproteobacteria	Rickettsiales			
OTU_1239	Bacteria	Proteobacteria	Alphaproteobacteria	Rickettsiales			
OTU_1259	Bacteria	Proteobacteria	Alphaproteobacteria	Rickettsiales			
OTU_1040	Bacteria	Proteobacteria	Alphaproteobacteria	Rickettsiales			
OTU_1001	Bacteria	Proteobacteria	Alphaproteobacteria	Rickettsiales			
OTU_713	Bacteria	Proteobacteria	Alphaproteobacteria	Sphingomonadales			
OTU_802	Bacteria	Proteobacteria	Alphaproteobacteria				
OTU_1130	Bacteria	Proteobacteria	Alphaproteobacteria				
OTU_1022	Bacteria	Proteobacteria	Alphaproteobacteria				
OTU_1072	Bacteria	Proteobacteria	Betaproteobacteria	Burkholderiales	Alcaligenaceae	Sutterella	
OTU_1183	Bacteria	Proteobacteria	Betaproteobacteria	Burkholderiales	Oxalobacteraceae	Ralstonia	
OTU_903	Bacteria	Proteobacteria	Betaproteobacteria	Burkholderiales	Oxalobacteraceae		
OTU_1121	Bacteria	Proteobacteria	Deltaproteobacteria	Bdellovibrionales	Bacteriovoracaceae		
OTU_1200	Bacteria	Proteobacteria	Deltaproteobacteria	Desulfobacterales	Desulfobulbaceae		
OTU_355	Bacteria	Proteobacteria	Deltaproteobacteria	Desulfovibrionales	Desulfovibrionaceae		
OTU_1076	Bacteria	Proteobacteria	Deltaproteobacteria	Myxococcales	OM27		
OTU_423	Bacteria	Proteobacteria	Deltaproteobacteria	PB19			
OTU_841	Bacteria	Proteobacteria	Deltaproteobacteria	Spirobacillales			
OTU_387	Bacteria	Proteobacteria	Deltaproteobacteria	Spirobacillales			
OTU_607	Bacteria	Proteobacteria	Deltaproteobacteria	Spirobacillales			

OTU_898	Bacteria	Proteobacteria	Deltaproteobacteria	Syntrophobacterales	Syntrophobacteraceae		
OTU_649	Bacteria	Proteobacteria	Deltaproteobacteria				
OTU_1124	Bacteria	Proteobacteria	Epsilonproteobacteria	Campylobacterales	Campylobacteraceae	Arcobacter	
OTU_910	Bacteria	Proteobacteria	Epsilonproteobacteria	Campylobacterales	Campylobacteraceae	Campylobacter	
OTU_204	Bacteria	Proteobacteria	Gammaproteobacteria	34P16			
OTU_733	Bacteria	Proteobacteria	Gammaproteobacteria	Aeromonadales	Succinivibrionaceae	Succinivibrio	
OTU_1010	Bacteria	Proteobacteria	Gammaproteobacteria	Alteromonadales	Alteromonadaceae		
OTU_592	Bacteria	Proteobacteria	Gammaproteobacteria	Alteromonadales	Alteromonadaceae		
OTU_757	Bacteria	Proteobacteria	Gammaproteobacteria	Alteromonadales	HTCC2188	HTCC	
OTU_68	Bacteria	Proteobacteria	Gammaproteobacteria	Alteromonadales	Shewanellaceae	Shewanella	algae
OTU_675	Bacteria	Proteobacteria	Gammaproteobacteria	Chromatiales	Halothiobacillaceae	Halothiobacillus	
OTU_1205	Bacteria	Proteobacteria	Gammaproteobacteria	Legionellales			
OTU_1033	Bacteria	Proteobacteria	Gammaproteobacteria	Legionellales			
OTU_190	Bacteria	Proteobacteria	Gammaproteobacteria	Oceanospirillales	Halomonadaceae	Halomonas	
OTU_67	Bacteria	Proteobacteria	Gammaproteobacteria	Oceanospirillales	Halomonadaceae		
OTU_786	Bacteria	Proteobacteria	Gammaproteobacteria	Oceanospirillales	Oleiphilaceae		
OTU_590	Bacteria	Proteobacteria	Gammaproteobacteria	Pseudomonadales	Moraxellaceae	Acinetobacter	rhizosphaerae
OTU_578	Bacteria	Proteobacteria	Gammaproteobacteria				
OTU_761	Bacteria	Proteobacteria					
OTU_814	Bacteria	Proteobacteria					
OTU_990	Bacteria	Proteobacteria					
OTU_732	Bacteria	Proteobacteria					
OTU_746	Bacteria	Proteobacteria					
OTU_863	Bacteria	Proteobacteria					
OTU_444	Bacteria	Proteobacteria					
OTU_1061	Bacteria	Proteobacteria					
OTU_944	Bacteria	Proteobacteria					
OTU_725	Bacteria	Proteobacteria					
OTU_779	Bacteria	Proteobacteria					
OTU_956	Bacteria	Proteobacteria					
OTU_73	Bacteria	Bacteroidetes	[Saprospirae]	[Saprospirales]	Saprospiraceae		
OTU_758	Bacteria	Bacteroidetes	Bacteroidia	Bacteroidales	[Paraprevotellaceae]	[Prevotella]	
OTU_1188	Bacteria	Bacteroidetes	Bacteroidia	Bacteroidales	[Paraprevotellaceae]	CF231	
OTU_888	Bacteria	Bacteroidetes	Bacteroidia	Bacteroidales	Prevotellaceae	Prevotella	
OTU_1202	Bacteria	Bacteroidetes	Bacteroidia	Bacteroidales	Prevotellaceae	Prevotella	
OTU_1187	Bacteria	Bacteroidetes	Bacteroidia	Bacteroidales	Prevotellaceae	Prevotella	
OTU_1031	Bacteria	Bacteroidetes	Bacteroidia	Bacteroidales	Prevotellaceae	Prevotella	
OTU_894	Bacteria	Bacteroidetes	Bacteroidia	Bacteroidales	S24-7		
OTU_558	Bacteria	Bacteroidetes	Bacteroidia	Bacteroidales			
OTU_398	Bacteria	Bacteroidetes	Flavobacteriia	Flavobacteriales	Flavobacteriaceae	Muricauda	
OTU_614	Bacteria	Bacteroidetes	Flavobacteriia	Flavobacteriales			
OTU_312	Bacteria	Bacteroidetes	Flavobacteriia	Flavobacteriales			
OTU_847	Bacteria	Bacteroidetes					

OTU_344	Bacteria	Actinobacteria	Acidimicrobiia	Acidimicrobiales			
OTU_316	Bacteria	Actinobacteria	Acidimicrobiia	Acidimicrobiales			
OTU_74	Bacteria	Actinobacteria	Acidimicrobiia	Acidimicrobiales			
OTU_1278	Bacteria	Actinobacteria	Acidimicrobiia	Acidimicrobiales			
OTU_309	Bacteria	Actinobacteria	Acidimicrobiia	Acidimicrobiales			
OTU_427	Bacteria	Actinobacteria	Acidimicrobiia	Acidimicrobiales			
OTU_1005	Bacteria	Actinobacteria	Acidimicrobiia	Acidimicrobiales			
OTU_302	Bacteria	Actinobacteria	Acidimicrobiia	Acidimicrobiales			
OTU_1237	Bacteria	Actinobacteria	Actinobacteria	Actinomycetales	Corynebacteriaceae	Corynebacterium	
OTU_556	Bacteria	Actinobacteria	Actinobacteria	Actinomycetales	Intrasporangiaceae		
OTU_1027	Bacteria	Actinobacteria	Actinobacteria	Actinomycetales	Microbacteriaceae	Candidatus Aquiluna	rubra
OTU_189	Bacteria	Actinobacteria	Actinobacteria	Actinomycetales	Pseudonocardiaceae		
OTU_80	Bacteria	Actinobacteria	Actinobacteria	Bifidobacteriales	Bifidobacteriaceae	Bifidobacterium	
OTU_411	Bacteria	Actinobacteria	Actinobacteria	Bifidobacteriales	Bifidobacteriaceae		
OTU_1169	Bacteria	Actinobacteria	Actinobacteria	Bifidobacteriales	Bifidobacteriaceae		
OTU_102	Bacteria	Actinobacteria	Actinobacteria	Bifidobacteriales	Bifidobacteriaceae		
OTU_967	Bacteria	Actinobacteria	Actinobacteria	Bifidobacteriales	Bifidobacteriaceae		
OTU_916	Bacteria	Actinobacteria	Coriobacteriia	Coriobacteriales	Coriobacteriaceae	Adlercreutzia	
OTU_1251	Bacteria	Actinobacteria	Coriobacteriia	Coriobacteriales	Coriobacteriaceae	Adlercreutzia	
OTU_855	Bacteria	Actinobacteria	Coriobacteriia	Coriobacteriales	Coriobacteriaceae	Adlercreutzia	
OTU_996	Bacteria	Actinobacteria	Coriobacteriia	Coriobacteriales	Coriobacteriaceae	Atopobium	
OTU_1269	Bacteria	Actinobacteria	Coriobacteriia	Coriobacteriales	Coriobacteriaceae	Atopobium	
OTU_1274	Bacteria	Actinobacteria	Coriobacteriia	Coriobacteriales	Coriobacteriaceae	Collinsella	aerofaciens
OTU_1059	Bacteria	Actinobacteria	Coriobacteriia	Coriobacteriales	Coriobacteriaceae		
OTU_793	Bacteria	Actinobacteria	Coriobacteriia	Coriobacteriales	Coriobacteriaceae		
OTU_1243	Bacteria	Actinobacteria	Coriobacteriia	Coriobacteriales	Coriobacteriaceae		
OTU_473	Bacteria	Actinobacteria	Coriobacteriia	Coriobacteriales	Coriobacteriaceae		
OTU_1161	Bacteria	Actinobacteria	Coriobacteriia	Coriobacteriales	Coriobacteriaceae		
OTU_1074	Bacteria	Actinobacteria	Coriobacteriia	Coriobacteriales	Coriobacteriaceae		
OTU_384	Bacteria	Actinobacteria	Coriobacteriia	Coriobacteriales	Coriobacteriaceae		
OTU_750	Bacteria	Actinobacteria	Coriobacteriia	Coriobacteriales	Coriobacteriaceae		
OTU_373	Bacteria	Actinobacteria	Coriobacteriia	Coriobacteriales	Coriobacteriaceae		
OTU_301	Bacteria	Firmicutes	Bacilli	Bacillales	Bacillaceae	Bacillus	cereus
OTU_510	Bacteria	Firmicutes	Bacilli	Lactobacillales	Aerococcaceae	Atopococcus	tabaci
OTU_1007	Bacteria	Firmicutes	Bacilli	Lactobacillales	Aerococcaceae		
OTU_557	Bacteria	Firmicutes	Bacilli	Lactobacillales	Streptococcaceae	Lactococcus	
OTU_563	Bacteria	Firmicutes	Clostridia	Clostridiales	[Mogibacteriaceae]	Mogibacterium	
OTU_1290	Bacteria	Firmicutes	Clostridia	Clostridiales	[Mogibacteriaceae]	Mogibacterium	
OTU_386	Bacteria	Firmicutes	Clostridia	Clostridiales	[Mogibacteriaceae]		
OTU_859	Bacteria	Firmicutes	Clostridia	Clostridiales	[Mogibacteriaceae]		
OTU_920	Bacteria	Firmicutes	Clostridia	Clostridiales	[Mogibacteriaceae]		
OTU_864	Bacteria	Firmicutes	Clostridia	Clostridiales	[Mogibacteriaceae]		
OTU_911	Bacteria	Firmicutes	Clostridia	Clostridiales	[Mogibacteriaceae]		

OTU_798	Bacteria	Firmicutes	Clostridia	Clostridiales	[Mogibacteriaceae]		
OTU_988	Bacteria	Firmicutes	Clostridia	Clostridiales	[Mogibacteriaceae]		
OTU_385	Bacteria	Firmicutes	Clostridia	Clostridiales	[Mogibacteriaceae]		
OTU_394	Bacteria	Firmicutes	Clostridia	Clostridiales	Christensenellaceae		
OTU_777	Bacteria	Firmicutes	Clostridia	Clostridiales	Christensenellaceae		
OTU_602	Bacteria	Firmicutes	Clostridia	Clostridiales	Christensenellaceae		
OTU_173	Bacteria	Firmicutes	Clostridia	Clostridiales	Clostridiaceae	Candidatus Arthromitus	
OTU_849	Bacteria	Firmicutes	Clostridia	Clostridiales	Clostridiaceae	Clostridium	
OTU_654	Bacteria	Firmicutes	Clostridia	Clostridiales	Clostridiaceae	Clostridium	
OTU_45	Bacteria	Firmicutes	Clostridia	Clostridiales	Clostridiaceae	Clostridium	butyricum
OTU_254	Bacteria	Firmicutes	Clostridia	Clostridiales	Clostridiaceae	Clostridium	perfringens
OTU_203	Bacteria	Firmicutes	Clostridia	Clostridiales	Clostridiaceae	Clostridium	
OTU_695	Bacteria	Firmicutes	Clostridia	Clostridiales	Clostridiaceae		
OTU_773	Bacteria	Firmicutes	Clostridia	Clostridiales	Dehalobacteriaceae	Dehalobacterium	
OTU_1134	Bacteria	Firmicutes	Clostridia	Clostridiales	Eubacteriaceae	Anaerofustis	
OTU_715	Bacteria	Firmicutes	Clostridia	Clostridiales	Lachnospiraceae	Butyrivibrio	
OTU_1083	Bacteria	Firmicutes	Clostridia	Clostridiales	Lachnospiraceae	Butyrivibrio	
OTU_644	Bacteria	Firmicutes	Clostridia	Clostridiales	Lachnospiraceae	Coprococcus	catus
OTU_623	Bacteria	Firmicutes	Clostridia	Clostridiales	Lachnospiraceae	Coprococcus	
OTU_772	Bacteria	Firmicutes	Clostridia	Clostridiales	Lachnospiraceae	Coprococcus	
OTU_1287	Bacteria	Firmicutes	Clostridia	Clostridiales	Lachnospiraceae	Coprococcus	
OTU_539	Bacteria	Firmicutes	Clostridia	Clostridiales	Lachnospiraceae	Coprococcus	
OTU_1189	Bacteria	Firmicutes	Clostridia	Clostridiales	Lachnospiraceae	Lachnospira	
OTU_271	Bacteria	Firmicutes	Clostridia	Clostridiales	Lachnospiraceae	Lachnospira	
OTU_1015	Bacteria	Firmicutes	Clostridia	Clostridiales	Lachnospiraceae		
OTU_1289	Bacteria	Firmicutes	Clostridia	Clostridiales	Lachnospiraceae		
OTU_928	Bacteria	Firmicutes	Clostridia	Clostridiales	Lachnospiraceae		
OTU_1038	Bacteria	Firmicutes	Clostridia	Clostridiales	Lachnospiraceae		
OTU_1184	Bacteria	Firmicutes	Clostridia	Clostridiales	Lachnospiraceae		
OTU_716	Bacteria	Firmicutes	Clostridia	Clostridiales	Lachnospiraceae		
OTU_1240	Bacteria	Firmicutes	Clostridia	Clostridiales	Lachnospiraceae		
OTU_1194	Bacteria	Firmicutes	Clostridia	Clostridiales	Lachnospiraceae		
OTU_999	Bacteria	Firmicutes	Clostridia	Clostridiales	Lachnospiraceae		
OTU_1049	Bacteria	Firmicutes	Clostridia	Clostridiales	Lachnospiraceae		
OTU_1177	Bacteria	Firmicutes	Clostridia	Clostridiales	Lachnospiraceae		
OTU_1036	Bacteria	Firmicutes	Clostridia	Clostridiales	Lachnospiraceae		
OTU_625	Bacteria	Firmicutes	Clostridia	Clostridiales	Lachnospiraceae		
OTU_689	Bacteria	Firmicutes	Clostridia	Clostridiales	Lachnospiraceae		
OTU_1133	Bacteria	Firmicutes	Clostridia	Clostridiales	Lachnospiraceae		
OTU_656	Bacteria	Firmicutes	Clostridia	Clostridiales	Lachnospiraceae		
OTU_618	Bacteria	Firmicutes	Clostridia	Clostridiales	Lachnospiraceae		
OTU_994	Bacteria	Firmicutes	Clostridia	Clostridiales	Lachnospiraceae		
OTU_1135	Bacteria	Firmicutes	Clostridia	Clostridiales	Lachnospiraceae		

OTU_852	Bacteria	Firmicutes	Clostridia	Clostridiales	Lachnospiraceae		
OTU_1056	Bacteria	Firmicutes	Clostridia	Clostridiales	Lachnospiraceae		
OTU_437	Bacteria	Firmicutes	Clostridia	Clostridiales	Lachnospiraceae		
OTU_1066	Bacteria	Firmicutes	Clostridia	Clostridiales	Lachnospiraceae		
OTU_1196	Bacteria	Firmicutes	Clostridia	Clostridiales	Lachnospiraceae		
OTU_904	Bacteria	Firmicutes	Clostridia	Clostridiales	Lachnospiraceae		
OTU_564	Bacteria	Firmicutes	Clostridia	Clostridiales	Lachnospiraceae		
OTU_351	Bacteria	Firmicutes	Clostridia	Clostridiales	Peptococcaceae	Peptococcus	
OTU_738	Bacteria	Firmicutes	Clostridia	Clostridiales	Ruminococcaceae	Butyricicoccus	pullicaecorum
OTU_909	Bacteria	Firmicutes	Clostridia	Clostridiales	Ruminococcaceae	Butyricicoccus	pullicaecorum
OTU_804	Bacteria	Firmicutes	Clostridia	Clostridiales	Ruminococcaceae	Oscillospira	
OTU_1272	Bacteria	Firmicutes	Clostridia	Clostridiales	Ruminococcaceae	Oscillospira	
OTU_328	Bacteria	Firmicutes	Clostridia	Clostridiales	Ruminococcaceae	Oscillospira	
OTU_787	Bacteria	Firmicutes	Clostridia	Clostridiales	Ruminococcaceae	Ruminococcus	
OTU_1105	Bacteria	Firmicutes	Clostridia	Clostridiales	Ruminococcaceae	Ruminococcus	
OTU_1127	Bacteria	Firmicutes	Clostridia	Clostridiales	Ruminococcaceae	Ruminococcus	
OTU_822	Bacteria	Firmicutes	Clostridia	Clostridiales	Ruminococcaceae	Ruminococcus	
OTU_1143	Bacteria	Firmicutes	Clostridia	Clostridiales	Ruminococcaceae	Ruminococcus	
OTU_1034	Bacteria	Firmicutes	Clostridia	Clostridiales	Ruminococcaceae	Ruminococcus	
OTU_726	Bacteria	Firmicutes	Clostridia	Clostridiales	Ruminococcaceae	Ruminococcus	
OTU_1012	Bacteria	Firmicutes	Clostridia	Clostridiales	Ruminococcaceae	Ruminococcus	
OTU_363	Bacteria	Firmicutes	Clostridia	Clostridiales	Ruminococcaceae	Ruminococcus	flavefaciens
OTU_279	Bacteria	Firmicutes	Clostridia	Clostridiales	Ruminococcaceae	Ruminococcus	
OTU_874	Bacteria	Firmicutes	Clostridia	Clostridiales	Ruminococcaceae	Ruminococcus	
OTU_826	Bacteria	Firmicutes	Clostridia	Clostridiales	Ruminococcaceae	Ruminococcus	
OTU_1125	Bacteria	Firmicutes	Clostridia	Clostridiales	Ruminococcaceae	Ruminococcus	
OTU_850	Bacteria	Firmicutes	Clostridia	Clostridiales	Ruminococcaceae	Ruminococcus	
OTU_781	Bacteria	Firmicutes	Clostridia	Clostridiales	Ruminococcaceae	Ruminococcus	
OTU_482	Bacteria	Firmicutes	Clostridia	Clostridiales	Ruminococcaceae	Ruminococcus	
OTU_1024	Bacteria	Firmicutes	Clostridia	Clostridiales	Ruminococcaceae	Ruminococcus	
OTU_1241	Bacteria	Firmicutes	Clostridia	Clostridiales	Ruminococcaceae		
OTU_340	Bacteria	Firmicutes	Clostridia	Clostridiales	Ruminococcaceae		
OTU_1224	Bacteria	Firmicutes	Clostridia	Clostridiales	Ruminococcaceae		
OTU_1048	Bacteria	Firmicutes	Clostridia	Clostridiales	Ruminococcaceae		
OTU_972	Bacteria	Firmicutes	Clostridia	Clostridiales	Ruminococcaceae		
OTU_307	Bacteria	Firmicutes	Clostridia	Clostridiales	Ruminococcaceae		
OTU_1046	Bacteria	Firmicutes	Clostridia	Clostridiales	Ruminococcaceae		
OTU_1197	Bacteria	Firmicutes	Clostridia	Clostridiales	Ruminococcaceae		
OTU_388	Bacteria	Firmicutes	Clostridia	Clostridiales	Ruminococcaceae		
OTU_577	Bacteria	Firmicutes	Clostridia	Clostridiales	Ruminococcaceae		
OTU_1091	Bacteria	Firmicutes	Clostridia	Clostridiales	Ruminococcaceae		
OTU_1128	Bacteria	Firmicutes	Clostridia	Clostridiales	Ruminococcaceae		
OTU_1231	Bacteria	Firmicutes	Clostridia	Clostridiales	Ruminococcaceae		

OTU_1098	Bacteria	Firmicutes	Clostridia	Clostridiales	Ruminococcaceae			
OTU_1221	Bacteria	Firmicutes	Clostridia	Clostridiales	Ruminococcaceae			
OTU_1145	Bacteria	Firmicutes	Clostridia	Clostridiales	Ruminococcaceae			
OTU_448	Bacteria	Firmicutes	Clostridia	Clostridiales	Ruminococcaceae			
OTU_1087	Bacteria	Firmicutes	Clostridia	Clostridiales	Ruminococcaceae			
OTU_1101	Bacteria	Firmicutes	Clostridia	Clostridiales	Ruminococcaceae			
OTU_937	Bacteria	Firmicutes	Clostridia	Clostridiales	Veillonellaceae	Phascolarctobacterium		
OTU_845	Bacteria	Firmicutes	Clostridia	Clostridiales				
OTU_843	Bacteria	Firmicutes	Clostridia	Clostridiales				
OTU_331	Bacteria	Firmicutes	Clostridia	Clostridiales				
OTU_1030	Bacteria	Firmicutes	Clostridia	Clostridiales				
OTU_867	Bacteria	Firmicutes	Clostridia	Clostridiales				
OTU_651	Bacteria	Firmicutes	Clostridia	Clostridiales				
OTU_371	Bacteria	Firmicutes	Clostridia	Clostridiales				
OTU_1209	Bacteria	Firmicutes	Clostridia	Clostridiales				
OTU_819	Bacteria	Firmicutes	Clostridia	Clostridiales				
OTU_472	Bacteria	Firmicutes	Clostridia	Clostridiales				
OTU_704	Bacteria	Firmicutes	Clostridia	Clostridiales				
OTU_839	Bacteria	Firmicutes	Clostridia	Clostridiales				
OTU_986	Bacteria	Firmicutes	Clostridia	Clostridiales				
OTU_828	Bacteria	Firmicutes	Clostridia	Clostridiales				
OTU_736	Bacteria	Firmicutes	Clostridia	Clostridiales				
OTU_766	Bacteria	Firmicutes	Clostridia					
OTU_938	Bacteria	Firmicutes	Erysipelotrichi	Erysipelotrichales	Erysipelotrichaceae	[Eubacterium]	biforme	
OTU_730	Bacteria	Firmicutes	Erysipelotrichi	Erysipelotrichales	Erysipelotrichaceae	Allobaculum		
OTU_791	Bacteria	Firmicutes	Erysipelotrichi	Erysipelotrichales	Erysipelotrichaceae	Allobaculum		
OTU_575	Bacteria	Firmicutes	Erysipelotrichi	Erysipelotrichales	Erysipelotrichaceae	Allobaculum		
OTU_945	Bacteria	Firmicutes	Erysipelotrichi	Erysipelotrichales	Erysipelotrichaceae	Allobaculum		
OTU_1097	Bacteria	Firmicutes	Erysipelotrichi	Erysipelotrichales	Erysipelotrichaceae	Bulleidia		
OTU_1110	Bacteria	Firmicutes	Erysipelotrichi	Erysipelotrichales	Erysipelotrichaceae	Bulleidia		
OTU_1050	Bacteria	Firmicutes	Erysipelotrichi	Erysipelotrichales	Erysipelotrichaceae	Bulleidia		
OTU_753	Bacteria	Firmicutes	Erysipelotrichi	Erysipelotrichales	Erysipelotrichaceae	Bulleidia	p-1630-c5	
OTU_441	Bacteria	Firmicutes	Erysipelotrichi	Erysipelotrichales	Erysipelotrichaceae	Clostridium	cocleatum	
OTU_811	Bacteria	Firmicutes	Erysipelotrichi	Erysipelotrichales	Erysipelotrichaceae	L7A_E11		
OTU_1154	Bacteria	Firmicutes	Erysipelotrichi	Erysipelotrichales	Erysipelotrichaceae	p-75-a5		
OTU_759	Bacteria	Firmicutes	Erysipelotrichi	Erysipelotrichales	Erysipelotrichaceae	p-75-a5		
OTU_1104	Bacteria	Firmicutes	Erysipelotrichi	Erysipelotrichales	Erysipelotrichaceae	Sharpea	azabuensis	
OTU_1011	Bacteria	Chloroflexi	Anaerolineae	Anaerolineales	Anaerolinaceae	SHD-231		
OTU_881	Bacteria	Cyanobacteria	Chloroplast	Chlorophyta				
OTU_673	Archaea	Euryarchaeota	Methanobacteria	Methanobacteriales	Methanobacteriaceae	Methanobrevibacter		
OTU_939	Archaea	Euryarchaeota	Methanobacteria	Methanobacteriales	Methanobacteriaceae	Methanobrevibacter		
OTU_432	Bacteria	Fusobacteria	Fusobacteriia	Fusobacteriales	Fusobacteriaceae	Fusobacterium		
OTU_784	Bacteria	Fusobacteria	Fusobacteriia	Fusobacteriales	Leptotrichiaceae	Leptotrichia		

OTU_901	Bacteria	Planctomycetes	C6	d113			
OTU_612	Bacteria	Planctomycetes	Planctomycetia	Pirellulales	Pirellulaceae	planctomycete	MS30D1
OTU_1035	Bacteria	Spirochaetes	Spirochaetes	Spirochaetales	Spirochaetaceae	Treponema	
OTU_534	Bacteria	Tenericutes	Mollicutes	Anaeroplasmatales	Anaeroplasmataceae	Anaeroplasma	
OTU_284	Bacteria	Tenericutes	Mollicutes	Mycoplasmatales	Mycoplasmataceae		
OTU_418	Bacteria	TM7	TM7-1				
OTU_964	Bacteria	TM7	TM7-3	CW040	F16		
OTU_825	Bacteria	TM7	TM7-3				
OTU_1118	Bacteria	Verrucomicrobia	Verrucomicrobiae	Verrucomicrobiales	Verrucomicrobiaceae		
OTU_483	Bacteria						
OTU_763	Unclassified						
OTU_824	Unclassified						
OTU_364	Unclassified						
OTU_1060	Bacteria						
OTU_1055	Bacteria						
OTU_325	Bacteria						
OTU_542	Bacteria						
OTU_862	Bacteria						
OTU_424	Bacteria						
OTU_952	Bacteria						
OTU_1291	Bacteria						
OTU_1164	Bacteria						
OTU_1213	Bacteria						
OTU_801	Bacteria						
OTU_1207	Bacteria						
OTU_541	Bacteria						
OTU_1226	Bacteria						
OTU_588	Bacteria						
OTU_953	Bacteria						
OTU_536	Bacteria						
OTU_930	Bacteria						
OTU_829	Bacteria						
OTU_681	Bacteria						
OTU_1280	Bacteria						
OTU_724	Unclassified						
OTU_599	Bacteria						
OTU_606	Bacteria						
OTU_1000	Bacteria						
OTU_647	Unclassified						
OTU_1080	Unclassified						
OTU_405	Bacteria						
OTU_322	Bacteria						
OTU_1267	Unclassified						

OTU_543	Bacteria
OTU_1065	Bacteria
OTU_980	Bacteria
OTU_245	Bacteria
OTU_992	Bacteria
OTU_720	Bacteria
OTU_493	Bacteria
OTU_333	Unclassified
OTU_1199	Bacteria
OTU_1142	Bacteria
OTU_183	Unclassified
OTU_984	Bacteria
OTU_889	Bacteria
OTU_1090	Bacteria
OTU_636	Bacteria
OTU_1045	Bacteria
OTU_566	Bacteria
OTU_806	Bacteria
OTU_692	Bacteria
OTU_1228	Bacteria
OTU_922	Bacteria
OTU_1151	Bacteria
OTU_1293	Bacteria
OTU_1122	Bacteria
OTU_1132	Bacteria
OTU_718	Unclassified
OTU_367	Bacteria
OTU_1109	Bacteria
OTU_1273	Bacteria
OTU_1185	Bacteria
OTU_812	Bacteria
OTU_723	Bacteria
OTU_1284	Bacteria
OTU_1215	Bacteria
OTU_1248	Bacteria
OTU_1159	Bacteria
OTU_1053	Bacteria
OTU_765	Bacteria
OTU_1265	Unclassified
OTU_1286	Bacteria
OTU_818	Bacteria
OTU_961	Bacteria
OTU_601	Unclassified

OTU_646	Bacteria						
OTU_1211	Bacteria						
OTU_1172	Bacteria						
OTU_982	Unclassified						
OTU_830	Bacteria						
OTU_805	Bacteria						
OTU_519	Bacteria						
OTU_435	Unclassified						
OTU_831	Bacteria						
OTU_827	Bacteria						
OTU_708	Bacteria						
OTU_857	Bacteria						
OTU_545	Unclassified						
OTU_1229	Bacteria						
OTU_109	Bacteria						
OTU_298	Bacteria						
OTU_1233	Unclassified						
OTU_709	Bacteria						
OTU_306	Bacteria						
OTU_774	Bacteria						
OTU_1255	Bacteria						
OTU_1220	Bacteria						
OTU_974	Bacteria						
OTU_1260	Bacteria						
OTU_1264	Bacteria						
OTU_1058	Bacteria						
OTU_747	Bacteria						
OTU_323	Bacteria						

CWS							
**OTUs**	**Kingdom**	**Phylum**	**Class**	**Order**	**Family**	**Genus**	**Specie**
OTU_420	Bacteria	Proteobacteria	Alphaproteobacteria	Rhodobacterales	Rhodobacteraceae	Rubellimicrobium	
OTU_1032	Bacteria	Proteobacteria	Alphaproteobacteria	Rickettsiales			
OTU_233	Bacteria	Proteobacteria	Alphaproteobacteria	Rickettsiales			
OTU_570	Bacteria	Proteobacteria	Alphaproteobacteria	Rickettsiales			
OTU_571	Bacteria	Proteobacteria	Alphaproteobacteria	Rickettsiales			
OTU_348	Bacteria	Proteobacteria	Alphaproteobacteria	Rickettsiales			
OTU_975	Bacteria	Proteobacteria	Alphaproteobacteria	Rickettsiales			
OTU_1258	Bacteria	Proteobacteria	Alphaproteobacteria	Rickettsiales			
OTU_91	Bacteria	Proteobacteria	Alphaproteobacteria	Sphingomonadales	Sphingomonadaceae	Kaistobacter	
OTU_1166	Bacteria	Proteobacteria	Alphaproteobacteria	Sphingomonadales	Sphingomonadaceae	Sphingomonas	
OTU_296	Bacteria	Proteobacteria	Betaproteobacteria	Burkholderiales	Alcaligenaceae		
OTU_178	Bacteria	Proteobacteria	Betaproteobacteria	Burkholderiales	Comamonadaceae		

OTU_131	Bacteria	Proteobacteria	Betaproteobacteria	Burkholderiales	Comamonadaceae			
OTU_856	Bacteria	Proteobacteria	Betaproteobacteria	Ellin6067				
OTU_172	Bacteria	Proteobacteria	Betaproteobacteria	Ellin6067				
OTU_218	Bacteria	Proteobacteria	Betaproteobacteria	Hydrogenophilales	Hydrogenophilaceae	Thiobacillus		
OTU_1193	Bacteria	Proteobacteria	Betaproteobacteria	Methylophilales	Methylophilaceae	Methylotenera	mobilis	
OTU_741	Bacteria	Proteobacteria	Betaproteobacteria	SC-I-84				
OTU_1168	Bacteria	Proteobacteria	Deltaproteobacteria	Bdellovibrionales	Bacteriovoracaceae	Bacteriovorax		
OTU_848	Bacteria	Proteobacteria	Deltaproteobacteria	Bdellovibrionales	Bacteriovoracaceae			
OTU_194	Bacteria	Proteobacteria	Deltaproteobacteria	BPC076				
OTU_365	Bacteria	Proteobacteria	Deltaproteobacteria	Desulfobacterales	Desulfobulbaceae			
OTU_755	Bacteria	Proteobacteria	Deltaproteobacteria	Desulfuromonadales	Geobacteraceae	Geobacter		
OTU_691	Bacteria	Proteobacteria	Deltaproteobacteria	Desulfuromonadales	Geobacteraceae	Geobacter		
OTU_719	Bacteria	Proteobacteria	Deltaproteobacteria	MIZ46				
OTU_395	Bacteria	Proteobacteria	Deltaproteobacteria	MIZ46				
OTU_267	Bacteria	Proteobacteria	Deltaproteobacteria	Myxococcales	Cystobacterineae			
OTU_303	Bacteria	Proteobacteria	Deltaproteobacteria	Myxococcales	Cystobacterineae			
OTU_147	Bacteria	Proteobacteria	Deltaproteobacteria	Myxococcales	Myxococcaceae	Anaeromyxobacter		
OTU_257	Bacteria	Proteobacteria	Deltaproteobacteria	Myxococcales				
OTU_594	Bacteria	Proteobacteria	Deltaproteobacteria	Myxococcales				
OTU_484	Bacteria	Proteobacteria	Deltaproteobacteria	Myxococcales				
OTU_291	Bacteria	Proteobacteria	Deltaproteobacteria	Myxococcales				
OTU_479	Bacteria	Proteobacteria	Deltaproteobacteria	Syntrophobacterales	Syntrophaceae			
OTU_126	Bacteria	Proteobacteria	Deltaproteobacteria					
OTU_507	Bacteria	Proteobacteria	Gammaproteobacteria	Alteromonadales	[Chromatiaceae]	Alishewanella		
OTU_391	Bacteria	Proteobacteria	Gammaproteobacteria	Alteromonadales	125ds10			
OTU_835	Bacteria	Proteobacteria	Gammaproteobacteria	Alteromonadales	Colwelliaceae	Thalassomonas		
OTU_752	Bacteria	Proteobacteria	Gammaproteobacteria	Alteromonadales	Idiomarinaceae	Pseudidiomarina		
OTU_282	Bacteria	Proteobacteria	Gammaproteobacteria	Chromatiales	Chromatiaceae	Thiocapsa		
OTU_1217	Bacteria	Proteobacteria	Gammaproteobacteria	Chromatiales	Chromatiaceae			
OTU_246	Bacteria	Proteobacteria	Gammaproteobacteria	Chromatiales				
OTU_133	Bacteria	Proteobacteria	Gammaproteobacteria	HOC36				
OTU_470	Bacteria	Proteobacteria	Gammaproteobacteria	Methylococcales	Methylococcaceae			
OTU_90	Bacteria	Proteobacteria	Gammaproteobacteria	Methylococcales				
OTU_1250	Bacteria	Proteobacteria	Gammaproteobacteria	Oceanospirillales	Oceanospirillaceae			
OTU_895	Bacteria	Proteobacteria	Gammaproteobacteria	Pseudomonadales	Moraxellaceae	Acinetobacter		
OTU_86	Bacteria	Proteobacteria	Gammaproteobacteria	Pseudomonadales	Pseudomonadaceae	Pseudomonas		
OTU_234	Bacteria	Proteobacteria	Gammaproteobacteria	Pseudomonadales	Pseudomonadaceae	Pseudomonas	stutzeri	
OTU_214	Bacteria	Proteobacteria	Gammaproteobacteria	Pseudomonadales	Pseudomonadaceae			
OTU_842	Bacteria	Proteobacteria	Gammaproteobacteria	Thiotrichales	Thiotrichaceae	Thiothrix		
OTU_255	Bacteria	Proteobacteria	Gammaproteobacteria	Xanthomonadales	Sinobacteraceae			
OTU_210	Bacteria	Proteobacteria	Gammaproteobacteria					
OTU_275	Bacteria	Proteobacteria	Gammaproteobacteria					
OTU_705	Bacteria	Proteobacteria						

OTU_1223	Bacteria	Bacteroidetes	[Rhodothermi]	[Rhodothermales]	Rhodothermaceae		
OTU_262	Bacteria	Bacteroidetes	Bacteroidia	Bacteroidales	Porphyromonadaceae	Paludibacter	
OTU_278	Bacteria	Bacteroidetes	Bacteroidia	Bacteroidales	Porphyromonadaceae	Parabacteroides	distasonis
OTU_352	Bacteria	Bacteroidetes	Bacteroidia	Bacteroidales	RF16		
OTU_390	Bacteria	Bacteroidetes	Bacteroidia	Bacteroidales	S24-7		
OTU_421	Bacteria	Bacteroidetes	Bacteroidia	Bacteroidales	SB-1		
OTU_235	Bacteria	Bacteroidetes	Bacteroidia	Bacteroidales			
OTU_426	Bacteria	Bacteroidetes	Bacteroidia	Bacteroidales			
OTU_221	Bacteria	Bacteroidetes	Flavobacteriia	Flavobacteriales	Cryomorphaceae		
OTU_439	Bacteria	Bacteroidetes	Flavobacteriia	Flavobacteriales	Flavobacteriaceae	Myroides	odoratimimus
OTU_533	Bacteria	Bacteroidetes	Flavobacteriia	Flavobacteriales			
OTU_884	Bacteria	Bacteroidetes	Sphingobacteriia	Sphingobacteriales	NS11-12		
OTU_547	Bacteria	Bacteroidetes					
OTU_1180	Bacteria	Bacteroidetes					
OTU_100	Bacteria	Actinobacteria	Acidimicrobiia	Acidimicrobiales	EB1017		
OTU_82	Bacteria	Actinobacteria	Acidimicrobiia	Acidimicrobiales	koll13		
OTU_329	Bacteria	Actinobacteria	Actinobacteria	Actinomycetales	Actinomycetaceae	Actinomyces	
OTU_508	Bacteria	Actinobacteria	Actinobacteria	Actinomycetales	Corynebacteriaceae	Corynebacterium	
OTU_216	Bacteria	Actinobacteria	Actinobacteria	Actinomycetales	Geodermatophilaceae		
OTU_341	Bacteria	Actinobacteria	OPB41				
OTU_292	Bacteria	Actinobacteria	Thermoleophilia	Gaiellales	Gaiellaceae		
OTU_400	Bacteria	Actinobacteria	Thermoleophilia	Gaiellales			
OTU_252	Bacteria	Actinobacteria	Thermoleophilia	Gaiellales			
OTU_790	Bacteria	Actinobacteria	Thermoleophilia	Gaiellales			
OTU_1071	Bacteria	Actinobacteria	Thermoleophilia				
OTU_171	Bacteria	Firmicutes	Bacilli	Bacillales	Bacillaceae		
OTU_281	Bacteria	Firmicutes	Bacilli	Bacillales	Bacillaceae		
OTU_518	Bacteria	Firmicutes	Bacilli	Bacillales	Bacillaceae		
OTU_336	Bacteria	Firmicutes	Bacilli	Lactobacillales	Aerococcaceae	Abiotrophia	
OTU_134	Bacteria	Firmicutes	Bacilli	Lactobacillales	Carnobacteriaceae		
OTU_206	Bacteria	Firmicutes	Clostridia	Clostridiales	[Mogibacteriaceae]	Anaerovorax	
OTU_466	Bacteria	Firmicutes	Clostridia	Clostridiales	[Tissierellaceae]	Peptoniphilus	
OTU_347	Bacteria	Firmicutes	Clostridia	Clostridiales	[Tissierellaceae]	Peptoniphilus	
OTU_927	Bacteria	Firmicutes	Clostridia	Clostridiales	Christensenellaceae		
OTU_645	Bacteria	Firmicutes	Clostridia	Clostridiales	Clostridiaceae	Caloramator	
OTU_495	Bacteria	Firmicutes	Clostridia	Clostridiales	Lachnospiraceae		
OTU_92	Bacteria	Firmicutes	Clostridia	Clostridiales	Ruminococcaceae		
OTU_702	Bacteria	Firmicutes	Clostridia	Clostridiales	Ruminococcaceae		
OTU_366	Bacteria	Firmicutes	Clostridia	Clostridiales	Ruminococcaceae		
OTU_504	Bacteria	Firmicutes	Clostridia	Clostridiales			
OTU_380	Bacteria	[Caldithrix]	KSB1	WM105			
OTU_196	Archaea	[Parvarchaeota]	[Parvarchaea]	YLA114			
OTU_96	Archaea	[Parvarchaeota]	[Parvarchaea]	YLA114			

OTU_268	Archaea	[Parvarchaeota]	[Parvarchaea]	YLA114		
OTU_650	Bacteria	[Thermi]	Deinococci	Deinococcales	Deinococcaceae	Deinococcus
OTU_174	Bacteria	AC1	SHA-114			
OTU_969	Bacteria	Acidobacteria	[Chloracidobacteria]	RB41		
OTU_505	Bacteria	Acidobacteria	Acidobacteria-6	iii1-15		
OTU_450	Bacteria	Chlamydiae	Chlamydiia	Chlamydiales		
OTU_207	Bacteria	Chlorobi	BSV26	PK329		
OTU_1088	Bacteria	Chloroflexi	Anaerolineae	Caldilineales	Caldilineaceae	Caldilinea
OTU_530	Bacteria	Chloroflexi	Anaerolineae	Caldilineales	Caldilineaceae	
OTU_455	Bacteria	Chloroflexi	Anaerolineae	SHA-20		
OTU_632	Bacteria	Chloroflexi	Anaerolineae	SJA-15		
OTU_529	Bacteria	Chloroflexi	Anaerolineae	SJA-15		
OTU_179	Bacteria	Chloroflexi	Anaerolineae	SJA-15		
OTU_297	Bacteria	Chloroflexi	Anaerolineae			
OTU_155	Bacteria	Chloroflexi	Ellin6529			
OTU_285	Bacteria	Chloroflexi	Ellin6529			
OTU_108	Bacteria	Cyanobacteria	Chloroplast	Chlorophyta	Chlamydomonadaceae	
OTU_611	Bacteria	Cyanobacteria	Chloroplast	Chlorophyta	Trebouxiophyceae	
OTU_121	Bacteria	Cyanobacteria	Chloroplast	Chlorophyta		
OTU_132	Bacteria	Cyanobacteria	Chloroplast	Stramenopiles		
OTU_124	Bacteria	Cyanobacteria	Chloroplast	Stramenopiles		
OTU_187	Bacteria	Cyanobacteria	Chloroplast	Stramenopiles		
OTU_456	Bacteria	Cyanobacteria				
OTU_399	Archaea	Euryarchaeota	Methanomicrobia	Methanosarcinales	Methanosaetaceae	Methanosaeta
OTU_265	Bacteria	Gemmatimonadetes	Gemmatimonadetes	KD8-87		
OTU_754	Bacteria	Gemmatimonadetes	Gemmatimonadetes			
OTU_266	Bacteria	Gemmatimonadetes	Gemmatimonadetes			
OTU_346	Bacteria	LCP-89	SAW1_B44			
OTU_354	Bacteria	Spirochaetes	Spirochaetes	Spirochaetales	Spirochaetaceae	
OTU_800	Bacteria	TM6	SJA-4			
OTU_205	Bacteria	Verrucomicrobia	[Pedosphaerae]	[Pedosphaerales]		
OTU_580	Bacteria	Verrucomicrobia	Opitutae	Opitutales	Opitutaceae	Opitutus
OTU_105	Bacteria	Verrucomicrobia	Verrucomicrobiae	Verrucomicrobiales	Verrucomicrobiaceae	Luteolibacter
OTU_99	Bacteria	WS3	PRR-12	Sediment-1		
OTU_412	Bacteria	WS5				
OTU_1108	Bacteria					
OTU_796	Bacteria					
OTU_1299	Bacteria					
OTU_934	Bacteria					
OTU_622	Bacteria					
OTU_1068	Unclassified					
OTU_523	Bacteria					
OTU_1103	Bacteria					

OTU_816	Bacteria						
OTU_664	Bacteria						
OTU_1004	Bacteria						
OTU_220	Bacteria						
OTU_288	Bacteria						
OTU_117	Bacteria						
OTU_152	Bacteria						
OTU_970	Bacteria						
OTU_965	Bacteria						
OTU_471	Bacteria						
OTU_1067	Bacteria						
OTU_464	Bacteria						
OTU_659	Bacteria						
OTU_810	Unclassified						
OTU_165	Bacteria						
OTU_269	Bacteria						
OTU_506	Bacteria						
OTU_560	Bacteria						
OTU_652	Bacteria						
OTU_591	Bacteria						
OTU_401	Bacteria						

BFT.W							
**OTUs**	**Kingdom**	**Phylum**	**Class**	**Order**	**Family**	**Genus**	**Specie**
OTU_1181	Bacteria	Proteobacteria	Alphaproteobacteria	BD7-3			
OTU_684	Bacteria	Proteobacteria	Alphaproteobacteria	Kiloniellales	Kiloniellaceae		
OTU_640	Bacteria	Proteobacteria	Alphaproteobacteria	Rhizobiales	Hyphomicrobiaceae		
OTU_1099	Bacteria	Proteobacteria	Alphaproteobacteria	Rhodospirillales	Rhodospirillaceae		
OTU_1254	Bacteria	Proteobacteria	Alphaproteobacteria	Rhodospirillales			
OTU_498	Bacteria	Proteobacteria	Alphaproteobacteria	Rickettsiales			
OTU_1019	Bacteria	Proteobacteria	Alphaproteobacteria	Rickettsiales			
OTU_1246	Bacteria	Proteobacteria	Alphaproteobacteria	Rickettsiales			
OTU_1078	Bacteria	Proteobacteria	Alphaproteobacteria				
OTU_792	Bacteria	Proteobacteria	Alphaproteobacteria				
OTU_1276	Bacteria	Proteobacteria	Betaproteobacteria	Nitrosomonadales	Nitrosomonadaceae		
OTU_620	Bacteria	Proteobacteria	Betaproteobacteria	SC-I-84			
OTU_851	Bacteria	Proteobacteria	Betaproteobacteria				
OTU_469	Bacteria	Proteobacteria	Deltaproteobacteria	Bdellovibrionales	Bacteriovoracaceae		
OTU_517	Bacteria	Proteobacteria	Deltaproteobacteria	Bdellovibrionales	Bacteriovoracaceae		
OTU_815	Bacteria	Proteobacteria	Deltaproteobacteria	Bdellovibrionales	Bdellovibrionaceae	Bdellovibrio	
OTU_1116	Bacteria	Proteobacteria	Deltaproteobacteria	Desulfuromonadales	Geobacteraceae	Geobacter	
OTU_976	Bacteria	Proteobacteria	Deltaproteobacteria	MIZ46			
OTU_731	Bacteria	Proteobacteria	Deltaproteobacteria	NB1-j	JTB38		

OTU_1186	Bacteria	Proteobacteria	Deltaproteobacteria	Spirobacillales			
OTU_481	Bacteria	Proteobacteria	Deltaproteobacteria	Spirobacillales			
OTU_1096	Bacteria	Proteobacteria	Deltaproteobacteria				
OTU_429	Bacteria	Proteobacteria	Gammaproteobacteria	[Marinicellales]	[Marinicellaceae]	Marinicella	
OTU_609	Bacteria	Proteobacteria	Gammaproteobacteria	[Marinicellales]	[Marinicellaceae]		
OTU_397	Bacteria	Proteobacteria	Gammaproteobacteria	Alteromonadales	Alteromonadaceae	BD2-13	
OTU_1230	Bacteria	Proteobacteria	Gammaproteobacteria	Alteromonadales	Alteromonadaceae	Marinobacter	
OTU_1216	Bacteria	Proteobacteria	Gammaproteobacteria	Alteromonadales	Alteromonadaceae		
OTU_492	Bacteria	Proteobacteria	Gammaproteobacteria	Alteromonadales	OM60		
OTU_433	Bacteria	Proteobacteria	Gammaproteobacteria	Enterobacteriales	Enterobacteriaceae	Leminorella	grimontii
OTU_1288	Bacteria	Proteobacteria	Gammaproteobacteria	Vibrionales	Vibrionaceae	Photobacterium	
OTU_565	Bacteria	Proteobacteria	Gammaproteobacteria				
OTU_767	Bacteria	Proteobacteria	Gammaproteobacteria				
OTU_756	Bacteria	Proteobacteria	TA18	CV90			
OTU_626	Bacteria	Proteobacteria	TA18	PHOS-HD29			
OTU_1077	Bacteria	Proteobacteria					
OTU_1114	Bacteria	Bacteroidetes	[Saprospirae]	[Saprospirales]	Saprospiraceae		
OTU_861	Bacteria	Bacteroidetes	[Saprospirae]	[Saprospirales]	Saprospiraceae		
OTU_973	Bacteria	Bacteroidetes	Bacteroidia	Bacteroidales	[Odoribacteraceae]	Odoribacter	
OTU_393	Bacteria	Bacteroidetes	Bacteroidia	Bacteroidales	Bacteroidaceae	Bacteroides	
OTU_1057	Bacteria	Bacteroidetes	Flavobacteriia	Flavobacteriales	[Weeksellaceae]		
OTU_745	Bacteria	Bacteroidetes	Flavobacteriia	Flavobacteriales	Flavobacteriaceae	Aquimarina	
OTU_410	Bacteria	Bacteroidetes	Flavobacteriia	Flavobacteriales	Flavobacteriaceae	Aquimarina	
OTU_1084	Bacteria	Bacteroidetes	Flavobacteriia	Flavobacteriales			
OTU_878	Bacteria	Bacteroidetes					
OTU_865	Bacteria	Bacteroidetes					
OTU_419	Bacteria	Actinobacteria	Acidimicrobiia	Acidimicrobiales			
OTU_840	Bacteria	Actinobacteria	Actinobacteria	Actinomycetales	Mycobacteriaceae	Mycobacterium	
OTU_1222	Bacteria	Firmicutes	Bacilli	Lactobacillales	Aerococcaceae	Facklamia	
OTU_1171	Bacteria	Firmicutes	Clostridia	Clostridiales	Clostridiaceae		
OTU_1006	Bacteria	Firmicutes	Clostridia	Clostridiales	Clostridiaceae		
OTU_943	Bacteria	Firmicutes	Clostridia	Clostridiales	Lachnospiraceae		
OTU_586	Bacteria	Firmicutes	Clostridia	Clostridiales	Lachnospiraceae		
OTU_775	Bacteria	Firmicutes	Clostridia	Clostridiales	Lachnospiraceae		
OTU_955	Bacteria	Firmicutes	Clostridia	Clostridiales	Lachnospiraceae		
OTU_1041	Bacteria	Firmicutes	Clostridia	Clostridiales	Ruminococcaceae	Oscillospira	
OTU_617	Bacteria	Firmicutes	Clostridia	Clostridiales	Ruminococcaceae	Ruminococcus	
OTU_1013	Bacteria	Firmicutes	Clostridia	Clostridiales	Ruminococcaceae		
OTU_971	Bacteria	Firmicutes	Clostridia	Clostridiales	Syntrophomonadaceae	Syntrophomonas	
OTU_1016	Bacteria	Firmicutes	Clostridia	Clostridiales	Veillonellaceae	Veillonella	
OTU_1257	Bacteria	Firmicutes	Clostridia	Clostridiales			
OTU_1113	Bacteria	Firmicutes	Clostridia	Clostridiales			
OTU_596	Bacteria	Firmicutes	Clostridia	Clostridiales			

OTU_619	Bacteria	Firmicutes	Clostridia	Clostridiales		
OTU_1073	Bacteria	Firmicutes	Clostridia	Clostridiales		
OTU_1123	Bacteria	Firmicutes	Clostridia	OPB54		
OTU_1292	Bacteria	Firmicutes	Erysipelotrichi	Erysipelotrichales	Erysipelotrichaceae	Erysipelothrix
OTU_981	Bacteria	Acidobacteria	Acidobacteria-6	iii1-15	RB40	
OTU_376	Bacteria	Acidobacteria	Sva0725	Sva0725		
OTU_375	Bacteria	Acidobacteria	Sva0725	Sva0725		
OTU_960	Bacteria	Armatimonadetes	[Fimbriimonadia]	[Fimbriimonadales]	[Fimbriimonadaceae]	
OTU_858	Bacteria	Caldithrix	Caldithrixae	Caldithrixales	BA059	
OTU_1136	Bacteria	Chloroflexi	TK17			
OTU_977	Bacteria	Planctomycetes	Phycisphaerae	Phycisphaerales		
OTU_1052	Bacteria	Planctomycetes	Planctomycetia	Pirellulales	Pirellulaceae	
OTU_880	Bacteria	Planctomycetes	Planctomycetia	Planctomycetales	Planctomycetaceae	Planctomyces
OTU_382	Bacteria	TM6	SJA-4			
OTU_868	Bacteria	TM7				
OTU_1162	Bacteria	Verrucomicrobia	[Pedosphaerae]			
OTU_583	Bacteria	Verrucomicrobia	Opitutae	[Pelagicoccales]	[Pelagicoccaceae]	Pelagicoccus
OTU_947	Bacteria	Verrucomicrobia	Verrucomicrobiae	Verrucomicrobiales	Verrucomicrobiaceae	MSBL3
OTU_886	Bacteria	Verrucomicrobia	Verrucomicrobiae	Verrucomicrobiales	Verrucomicrobiaceae	
OTU_860	Bacteria					
OTU_932	Unclassified					
OTU_987	Bacteria					
OTU_1140	Unclassified					
OTU_954	Unclassified					
OTU_946	Unclassified					
OTU_330	Bacteria					
OTU_1214	Bacteria					
OTU_1294	Bacteria					
OTU_332	Bacteria					
OTU_699	Bacteria					
OTU_1302	Bacteria					
OTU_1295	Bacteria					
OTU_933	Bacteria					
OTU_778	Bacteria					
OTU_785	Unclassified					
OTU_1054	Unclassified					
OTU_1262	Bacteria					
OTU_576	Unclassified					
OTU_1208	Bacteria					
OTU_1277	Bacteria					
OTU_776	Bacteria					
OTU_666	Bacteria					
OTU_948	Unclassified					

OTU_896	Bacteria						
OTU_1179	Bacteria						
OTU_177	Bacteria						
OTU_905	Bacteria						
OTU_1075	Bacteria						
OTU_378	Bacteria						

CWS.W							
**OTUs**	**Kingdom**	**Phylum**	**Class**	**Order**	**Family**	**Genus**	**Specie**
OTU_993	Bacteria	Proteobacteria	Alphaproteobacteria	BD7-3			
OTU_742	Bacteria	Proteobacteria	Alphaproteobacteria	Caulobacterales	Caulobacteraceae	Phenylobacterium	
OTU_1297	Bacteria	Proteobacteria	Alphaproteobacteria	Rhizobiales	Bradyrhizobiaceae	Balneimonas	
OTU_1235	Bacteria	Proteobacteria	Alphaproteobacteria	Rhodospirillales	Acetobacteraceae		
OTU_1174	Bacteria	Proteobacteria	Alphaproteobacteria	Rhodospirillales			
OTU_891	Bacteria	Proteobacteria	Alphaproteobacteria	Rickettsiales			
OTU_717	Bacteria	Proteobacteria	Alphaproteobacteria	Rickettsiales			
OTU_1227	Bacteria	Proteobacteria	Alphaproteobacteria	Rickettsiales			
OTU_1245	Bacteria	Proteobacteria	Alphaproteobacteria	Rickettsiales			
OTU_983	Bacteria	Proteobacteria	Alphaproteobacteria	Rickettsiales			
OTU_1044	Bacteria	Proteobacteria	Alphaproteobacteria				
OTU_902	Bacteria	Proteobacteria	Alphaproteobacteria				
OTU_764	Bacteria	Proteobacteria	Betaproteobacteria	Neisseriales	Neisseriaceae	Vitreoscilla	
OTU_116	Bacteria	Proteobacteria	Deltaproteobacteria	Bdellovibrionales	Bacteriovoracaceae	Bacteriovorax	
OTU_248	Bacteria	Proteobacteria	Deltaproteobacteria	Bdellovibrionales	Bacteriovoracaceae		
OTU_1092	Bacteria	Proteobacteria	Deltaproteobacteria	Desulfobacterales	Desulfobulbaceae	Desulfobulbus	
OTU_1023	Bacteria	Proteobacteria	Deltaproteobacteria	Desulfobacterales	Desulfobulbaceae		
OTU_231	Bacteria	Proteobacteria	Deltaproteobacteria	Desulfobacterales	Desulfobulbaceae		
OTU_549	Bacteria	Proteobacteria	Deltaproteobacteria	Desulfovibrionales	Desulfovibrionaceae	Desulfovibrio	
OTU_908	Bacteria	Proteobacteria	Deltaproteobacteria	Desulfovibrionales	Desulfovibrionaceae		
OTU_1026	Bacteria	Proteobacteria	Deltaproteobacteria	GMD14H09			
OTU_1079	Bacteria	Proteobacteria	Deltaproteobacteria				
OTU_236	Bacteria	Proteobacteria	Gammaproteobacteria	Alteromonadales			
OTU_1131	Bacteria	Proteobacteria	Gammaproteobacteria	Enterobacteriales	Enterobacteriaceae	Serratia	symbiotica
OTU_979	Bacteria	Proteobacteria	Gammaproteobacteria	Enterobacteriales	Enterobacteriaceae		
OTU_780	Bacteria	Proteobacteria	Gammaproteobacteria	Legionellales	Legionellaceae		
OTU_1129	Bacteria	Proteobacteria	Gammaproteobacteria				
OTU_1266	Bacteria	Proteobacteria					
OTU_683	Bacteria	Proteobacteria					
OTU_872	Bacteria	Proteobacteria					
OTU_1086	Bacteria	Proteobacteria					
OTU_1111	Bacteria	Proteobacteria					
OTU_197	Bacteria	Bacteroidetes	[Saprospirae]	[Saprospirales]	Saprospiraceae		
OTU_669	Bacteria	Bacteroidetes	[Saprospirae]	[Saprospirales]	Saprospiraceae		

OTU_112	Bacteria	Bacteroidetes	Bacteroidia	Bacteroidales	Marinilabiaceae		
OTU_567	Bacteria	Bacteroidetes	Bacteroidia	Bacteroidales	Marinilabiaceae		
OTU_295	Bacteria	Bacteroidetes	Bacteroidia	Bacteroidales	Marinilabiaceae		
OTU_1144	Bacteria	Bacteroidetes	Bacteroidia	Bacteroidales	Porphyromonadaceae	Paludibacter	
OTU_1281	Bacteria	Bacteroidetes	Bacteroidia	Bacteroidales	S24-7		
OTU_357	Bacteria	Bacteroidetes	Bacteroidia	Bacteroidales			
OTU_1148	Bacteria	Bacteroidetes	Cytophagia	Cytophagales	Flammeovirgaceae	JTB248	
OTU_1165	Bacteria	Bacteroidetes	Sphingobacteriia	Sphingobacteriales			
OTU_443	Bacteria	Bacteroidetes					
OTU_1256	Bacteria	Bacteroidetes					
OTU_527	Bacteria	Bacteroidetes					
OTU_1212	Bacteria	Actinobacteria	Acidimicrobiia	Acidimicrobiales			
OTU_551	Bacteria	Actinobacteria	Acidimicrobiia	Acidimicrobiales			
OTU_722	Bacteria	Actinobacteria	Actinobacteria	Actinomycetales	Corynebacteriaceae	Corynebacterium	
OTU_912	Bacteria	Actinobacteria	Rubrobacteria	Rubrobacterales	Rubrobacteraceae	Rubrobacter	
OTU_554	Bacteria	Actinobacteria	Rubrobacteria	Rubrobacterales	Rubrobacteraceae		
OTU_949	Bacteria	Actinobacteria	Thermoleophilia	Gaiellales	Gaiellaceae		
OTU_672	Bacteria	Actinobacteria	Thermoleophilia	Gaiellales	Gaiellaceae		
OTU_425	Bacteria	Firmicutes	Bacilli	Bacillales	Bacillaceae	Bacillus	
OTU_55	Bacteria	Firmicutes	Clostridia	Clostridiales	[Acidaminobacteraceae]	Fusibacter	
OTU_409	Bacteria	Firmicutes	Clostridia	Clostridiales	[Acidaminobacteraceae]	Fusibacter	
OTU_356	Bacteria	Firmicutes	Clostridia	Clostridiales	[Acidaminobacteraceae]	WH1-8	
OTU_1170	Bacteria	Firmicutes	Clostridia	Clostridiales	[Mogibacteriaceae]	Anaerovorax	
OTU_1043	Bacteria	Firmicutes	Clostridia	Clostridiales	[Tissierellaceae]	Sporanaerobacter	
OTU_1253	Bacteria	Firmicutes	Clostridia	Clostridiales	Christensenellaceae		
OTU_1176	Bacteria	Firmicutes	Clostridia	Clostridiales	Christensenellaceae		
OTU_1126	Bacteria	Firmicutes	Clostridia	Clostridiales	Clostridiaceae		
OTU_998	Bacteria	Firmicutes	Clostridia	Clostridiales	Eubacteriaceae	eudoramibacter_Eubacterium	
OTU_966	Bacteria	Firmicutes	Clostridia	Clostridiales	Lachnospiraceae		
OTU_1282	Bacteria	Firmicutes	Clostridia	Clostridiales	Lachnospiraceae		
OTU_1153	Bacteria	Firmicutes	Clostridia	Clostridiales	Lachnospiraceae		
OTU_1139	Bacteria	Firmicutes	Clostridia	Clostridiales	Peptostreptococcaceae	Peptostreptococcus	
OTU_1020	Bacteria	Firmicutes	Clostridia	Clostridiales			
OTU_885	Bacteria	Firmicutes	Clostridia	Clostridiales			
OTU_995	Bacteria	Firmicutes	Clostridia	Clostridiales			
OTU_1191	Bacteria	Firmicutes	Clostridia	Natranaerobiales	Anaerobrancaceae	Dethiobacter	
OTU_621	Bacteria	Acidobacteria	Acidobacteria-6	iii1-15			
OTU_256	Bacteria	Chlorobi	SJA-28				
OTU_740	Bacteria	Chloroflexi	Anaerolineae	Anaerolineales	Anaerolinaceae	WCHB1-05	
OTU_585	Bacteria	Chloroflexi	Anaerolineae	S0208			
OTU_735	Bacteria	Chloroflexi	Anaerolineae				
OTU_270	Bacteria	Fibrobacteres	TG3	TG3-2			
OTU_59	Bacteria	Fusobacteria	Fusobacteriia	Fusobacteriales	Fusobacteriaceae	Cetobacterium	somerae

OTU_540	Bacteria	Fusobacteria	Fusobacteriia	Fusobacteriales	Fusobacteriaceae	Fusobacterium
OTU_202	Bacteria	Fusobacteria	Fusobacteriia	Fusobacteriales	Fusobacteriaceae	Propionigenium
OTU_873	Bacteria	Gemmatimonadetes	Gemmatimonadetes			
OTU_957	Bacteria	Nitrospirae	Nitrospira	Nitrospirales	Apr-29	
OTU_989	Bacteria	Nitrospirae	Nitrospira	Nitrospirales	hermodesulfovibrionaceae]	
OTU_846	Bacteria	OP9	JS1	BA021		
OTU_396	Bacteria	Tenericutes	CK-1C4-19			
OTU_1300	Bacteria	TM7				
OTU_1247	Bacteria					
OTU_581	Bacteria					
OTU_985	Bacteria					
OTU_480	Unclassified					
OTU_1115	Unclassified					
OTU_1150	Bacteria					
OTU_1252	Bacteria					
OTU_1175	Bacteria					
OTU_728	Bacteria					
OTU_678	Unclassified					
OTU_1002	Unclassified					
OTU_1037	Bacteria					
OTU_658	Unclassified					
OTU_877	Bacteria					
OTU_788	Bacteria					
OTU_1051	Bacteria					
OTU_1261	Bacteria					
OTU_680	Bacteria					
OTU_935	Bacteria					
OTU_929	Bacteria					
OTU_406	Bacteria					
OTU_462	Bacteria					
OTU_568	Bacteria					
OTU_502	Bacteria					
OTU_1285	Bacteria					
OTU_525	Bacteria					
OTU_522	Bacteria					
OTU_613	Bacteria					
OTU_1238	Unclassified					
OTU_1069	Bacteria					
OTU_887	Bacteria					
OTU_1137	Bacteria					
OTU_1063	Bacteria					
OTU_1204	Bacteria					
OTU_552	Bacteria					

OTU_631	Unclassified						

BFT.W x CWS.W							
**OTUs**	**Kingdom**	**Phylum**	**Class**	**Order**	**Family**	**Genus**	**Specie**
OTU_449	Bacteria	Proteobacteria	Alphaproteobacteria	BD7-3			
OTU_407	Bacteria	Proteobacteria	Alphaproteobacteria	Rhizobiales	Brucellaceae		
OTU_239	Bacteria	Proteobacteria	Alphaproteobacteria	Rhizobiales	Cohaesibacteraceae	Cohaesibacter	
OTU_883	Bacteria	Proteobacteria	Alphaproteobacteria	Rhizobiales	Hyphomicrobiaceae	Hyphomicrobium	
OTU_485	Bacteria	Proteobacteria	Alphaproteobacteria	Rhizobiales			
OTU_897	Bacteria	Proteobacteria	Alphaproteobacteria	Rhizobiales			
OTU_795	Bacteria	Proteobacteria	Alphaproteobacteria	Rhodobacterales	Hyphomonadaceae		
OTU_1120	Bacteria	Proteobacteria	Alphaproteobacteria	Rhodobacterales	Hyphomonadaceae		
OTU_392	Bacteria	Proteobacteria	Alphaproteobacteria	Rhodospirillales	Rhodospirillaceae		
OTU_809	Bacteria	Proteobacteria	Alphaproteobacteria	Rhodospirillales	Rhodospirillaceae		
OTU_807	Bacteria	Proteobacteria	Alphaproteobacteria	Rhodospirillales	Rhodospirillaceae		
OTU_1149	Bacteria	Proteobacteria	Alphaproteobacteria	Rhodospirillales	Rhodospirillaceae		
OTU_512	Bacteria	Proteobacteria	Alphaproteobacteria	Rhodospirillales	Rhodospirillaceae		
OTU_823	Bacteria	Proteobacteria	Alphaproteobacteria	Rhodospirillales	Rhodospirillaceae	Phaeospirillum	
OTU_1160	Bacteria	Proteobacteria	Alphaproteobacteria	Rhodospirillales	Rhodospirillaceae		
OTU_597	Bacteria	Proteobacteria	Alphaproteobacteria	Rhodospirillales			
OTU_1219	Bacteria	Proteobacteria	Alphaproteobacteria				
OTU_408	Bacteria	Proteobacteria	Betaproteobacteria	Burkholderiales	Alcaligenaceae	Alcaligenes	faecalis
OTU_193	Bacteria	Proteobacteria	Betaproteobacteria	Burkholderiales	Alcaligenaceae		
OTU_500	Bacteria	Proteobacteria	Betaproteobacteria	Burkholderiales	Comamonadaceae	Rubrivivax	
OTU_1047	Bacteria	Proteobacteria	Betaproteobacteria	Burkholderiales	Comamonadaceae		
OTU_923	Bacteria	Proteobacteria	Betaproteobacteria	Burkholderiales	Comamonadaceae	Comamonas	
OTU_438	Bacteria	Proteobacteria	Betaproteobacteria	Nitrosomonadales	Nitrosomonadaceae		
OTU_628	Bacteria	Proteobacteria	Betaproteobacteria	Nitrosomonadales	Nitrosomonadaceae		
OTU_199	Bacteria	Proteobacteria	Betaproteobacteria	Nitrosomonadales	Nitrosomonadaceae		
OTU_129	Bacteria	Proteobacteria	Betaproteobacteria	Rhodocyclales	Rhodocyclaceae	Dok59	
OTU_789	Bacteria	Proteobacteria	Betaproteobacteria	Rhodocyclales	Rhodocyclaceae	Methyloversatilis	
OTU_963	Bacteria	Proteobacteria	Betaproteobacteria	SBla14			
OTU_627	Bacteria	Proteobacteria	Deltaproteobacteria	Desulfovibrionales	Desulfovibrionaceae		
OTU_559	Bacteria	Proteobacteria	Deltaproteobacteria	Desulfuromonadales	Geobacteraceae	Geobacter	
OTU_584	Bacteria	Proteobacteria	Deltaproteobacteria	DTB120			
OTU_166	Bacteria	Proteobacteria	Deltaproteobacteria	Myxococcales	Haliangiaceae		
OTU_389	Bacteria	Proteobacteria	Deltaproteobacteria	Myxococcales	OM27		
OTU_440	Bacteria	Proteobacteria	Deltaproteobacteria	Myxococcales			
OTU_569	Bacteria	Proteobacteria	Deltaproteobacteria	Myxococcales			
OTU_941	Bacteria	Proteobacteria	Deltaproteobacteria	Myxococcales			
OTU_1152	Bacteria	Proteobacteria	Deltaproteobacteria	NB1-j			
OTU_1210	Bacteria	Proteobacteria	Deltaproteobacteria	Syntrophobacterales	Syntrophobacteraceae		
OTU_272	Bacteria	Proteobacteria	Deltaproteobacteria				

OTU_907	Bacteria	Proteobacteria	Epsilonproteobacteria	Campylobacterales	Helicobacteraceae	Helicobacter	
OTU_931	Bacteria	Proteobacteria	Gammaproteobacteria	Pseudomonadales	Moraxellaceae	Acinetobacter	
OTU_600	Bacteria	Proteobacteria	Gammaproteobacteria	Pseudomonadales	Pseudomonadaceae		
OTU_215	Bacteria	Proteobacteria	Gammaproteobacteria	Pseudomonadales	Pseudomonadaceae		
OTU_624	Bacteria	Proteobacteria	Gammaproteobacteria	Pseudomonadales	Pseudomonadaceae	Pseudomonas	
OTU_211	Bacteria	Proteobacteria	Gammaproteobacteria	Thiotrichales	Piscirickettsiaceae		
OTU_164	Bacteria	Proteobacteria	Gammaproteobacteria	Xanthomonadales	Xanthomonadaceae		
OTU_875	Bacteria	Proteobacteria	Gammaproteobacteria				
OTU_238	Bacteria	Proteobacteria	Gammaproteobacteria				
OTU_232	Bacteria	Bacteroidetes	[Saprospirae]	[Saprospirales]	Saprospiraceae		
OTU_460	Bacteria	Bacteroidetes	[Saprospirae]	[Saprospirales]	Saprospiraceae		
OTU_573	Bacteria	Bacteroidetes	Bacteroidia	Bacteroidales	[Odoribacteraceae]	Odoribacter	
OTU_537	Bacteria	Bacteroidetes	Bacteroidia	Bacteroidales	[Paraprevotellaceae]	[Prevotella]	
OTU_593	Bacteria	Bacteroidetes	Bacteroidia	Bacteroidales	Bacteroidaceae	Bacteroides	coprosuis
OTU_729	Bacteria	Bacteroidetes	Bacteroidia	Bacteroidales	Bacteroidaceae	Bacteroides	
OTU_383	Bacteria	Bacteroidetes	Bacteroidia	Bacteroidales	Bacteroidaceae	Bacteroides	
OTU_668	Bacteria	Bacteroidetes	Bacteroidia	Bacteroidales	Bacteroidaceae	Bacteroides	
OTU_808	Bacteria	Bacteroidetes	Bacteroidia	Bacteroidales	Porphyromonadaceae		
OTU_524	Bacteria	Bacteroidetes	Bacteroidia	Bacteroidales	Porphyromonadaceae		
OTU_968	Bacteria	Bacteroidetes	Bacteroidia	Bacteroidales	Porphyromonadaceae	Paludibacter	
OTU_739	Bacteria	Bacteroidetes	Bacteroidia	Bacteroidales	Porphyromonadaceae		
OTU_817	Bacteria	Bacteroidetes	Bacteroidia	Bacteroidales	Porphyromonadaceae		
OTU_561	Bacteria	Bacteroidetes	Bacteroidia	Bacteroidales	Rikenellaceae		
OTU_794	Bacteria	Bacteroidetes	Bacteroidia	Bacteroidales	Rikenellaceae		
OTU_694	Bacteria	Bacteroidetes	Bacteroidia	Bacteroidales	Rikenellaceae		
OTU_710	Bacteria	Bacteroidetes	Bacteroidia	Bacteroidales	S24-7		
OTU_1141	Bacteria	Bacteroidetes	Bacteroidia	Bacteroidales	S24-7		
OTU_1195	Bacteria	Bacteroidetes	Bacteroidia	Bacteroidales	S24-7		
OTU_635	Bacteria	Bacteroidetes	Bacteroidia	Bacteroidales	S24-7		
OTU_1094	Bacteria	Bacteroidetes	Bacteroidia	Bacteroidales	S24-7		
OTU_422	Bacteria	Bacteroidetes	Bacteroidia	Bacteroidales			
OTU_1283	Bacteria	Bacteroidetes	Cytophagia	Cytophagales	Cytophagaceae		
OTU_572	Bacteria	Bacteroidetes	Cytophagia	Cytophagales	Cytophagaceae		
OTU_803	Bacteria	Bacteroidetes	Flavobacteriia	Flavobacteriales	Cryomorphaceae	Cryomorpha	
OTU_343	Bacteria	Bacteroidetes	Flavobacteriia	Flavobacteriales	Cryomorphaceae		
OTU_749	Bacteria	Bacteroidetes	Flavobacteriia	Flavobacteriales	Flavobacteriaceae	Psychroserpens	
OTU_871	Bacteria	Actinobacteria	KIST-JJY010				
OTU_744	Bacteria	Firmicutes	Bacilli	Turicibacterales	Turicibacteraceae	Turicibacter	
OTU_882	Bacteria	Firmicutes	Clostridia	Clostridiales	[Tissierellaceae]	Sedimentibacter	
OTU_639	Bacteria	Firmicutes	Clostridia	Clostridiales	[Tissierellaceae]	GW-34	
OTU_942	Bacteria	Firmicutes	Clostridia	Clostridiales	[Tissierellaceae]		
OTU_544	Bacteria	Firmicutes	Clostridia	Clostridiales	[Tissierellaceae]	Tepidimicrobium	
OTU_667	Bacteria	Firmicutes	Clostridia	Clostridiales	[Tissierellaceae]		

OTU_1008	Bacteria	Firmicutes	Clostridia	Clostridiales	Caldicoprobacteraceae	Caldicoprobacter
OTU_486	Bacteria	Firmicutes	Clostridia	Clostridiales	Clostridiaceae	Clostridium
OTU_799	Bacteria	Firmicutes	Clostridia	Clostridiales	Clostridiaceae	Clostridium
OTU_925	Bacteria	Firmicutes	Clostridia	Clostridiales	Clostridiaceae	Clostridium
OTU_641	Bacteria	Firmicutes	Clostridia	Clostridiales	Clostridiaceae	Clostridium
OTU_1270	Bacteria	Firmicutes	Clostridia	Clostridiales	Clostridiaceae	Clostridium
OTU_892	Bacteria	Firmicutes	Clostridia	Clostridiales	Gracilibacteraceae	
OTU_546	Bacteria	Firmicutes	Clostridia	Clostridiales	Gracilibacteraceae	Lutispora
OTU_978	Bacteria	Firmicutes	Clostridia	Clostridiales	Lachnospiraceae	
OTU_516	Bacteria	Firmicutes	Clostridia	Clostridiales	Lachnospiraceae	
OTU_1003	Bacteria	Firmicutes	Clostridia	Clostridiales	Lachnospiraceae	
OTU_832	Bacteria	Firmicutes	Clostridia	Clostridiales	Lachnospiraceae	
OTU_1275	Bacteria	Firmicutes	Clostridia	Clostridiales	Lachnospiraceae	
OTU_703	Bacteria	Firmicutes	Clostridia	Clostridiales	Lachnospiraceae	
OTU_451	Bacteria	Firmicutes	Clostridia	Clostridiales	Peptococcaceae	Pelotomaculum
OTU_671	Bacteria	Firmicutes	Clostridia	Clostridiales	Peptococcaceae	
OTU_962	Bacteria	Firmicutes	Clostridia	Clostridiales	Peptococcaceae	Pelotomaculum
OTU_1119	Bacteria	Firmicutes	Clostridia	Clostridiales	Peptococcaceae	Pelotomaculum
OTU_1206	Bacteria	Firmicutes	Clostridia	Clostridiales	Ruminococcaceae	
OTU_434	Bacteria	Firmicutes	Clostridia	Clostridiales	Ruminococcaceae	Oscillospira
OTU_714	Bacteria	Firmicutes	Clostridia	Clostridiales	Ruminococcaceae	Ruminococcus
OTU_454	Bacteria	Firmicutes	Clostridia	Clostridiales	Ruminococcaceae	
OTU_315	Bacteria	Firmicutes	Clostridia	Clostridiales	Ruminococcaceae	
OTU_924	Bacteria	Firmicutes	Clostridia	Clostridiales	Ruminococcaceae	
OTU_1242	Bacteria	Firmicutes	Clostridia	Clostridiales	Ruminococcaceae	Ruminococcus
OTU_690	Bacteria	Firmicutes	Clostridia	Clostridiales	Ruminococcaceae	
OTU_610	Bacteria	Firmicutes	Clostridia	Clostridiales	Syntrophomonadaceae	Syntrophomonas
OTU_1156	Bacteria	Firmicutes	Clostridia	Clostridiales	Syntrophomonadaceae	Syntrophomonas
OTU_321	Bacteria	Firmicutes	Clostridia	Clostridiales		
OTU_436	Bacteria	Firmicutes	Clostridia	Clostridiales		
OTU_1296	Bacteria	Firmicutes	Clostridia	Clostridiales		
OTU_616	Bacteria	Firmicutes	Clostridia	Clostridiales		
OTU_1081	Bacteria	Firmicutes	Clostridia	Clostridiales		
OTU_478	Bacteria	Firmicutes	Clostridia	Clostridiales		
OTU_553	Bacteria	Firmicutes	Clostridia	Clostridiales		
OTU_251	Bacteria	Firmicutes	Clostridia	Clostridiales		
OTU_1158	Bacteria	Firmicutes	Clostridia	Clostridiales		
OTU_1279	Bacteria	Firmicutes	Clostridia	Clostridiales		
OTU_821	Bacteria	Firmicutes	Clostridia	Clostridiales		
OTU_1271	Bacteria	Firmicutes	Clostridia	Clostridiales		
OTU_771	Bacteria	Firmicutes	Clostridia	Halanaerobiales	Halanaerobiaceae	
OTU_1147	Bacteria	Firmicutes	Clostridia	MBA08		
OTU_797	Bacteria	Firmicutes	Clostridia	Natranaerobiales	Anaerobrancaceae	Dethiobacter

OTU_833	Bacteria	Firmicutes	Clostridia	Natranaerobiales	ML1228J-1		
OTU_1218	Bacteria	Firmicutes	Clostridia	OPB54			
OTU_834	Bacteria	Firmicutes	Clostridia	SHA-98			
OTU_700	Bacteria	Firmicutes	Erysipelotrichi	Erysipelotrichales	Erysipelotrichaceae	Erysipelothrix	
OTU_1112	Bacteria	Firmicutes	Erysipelotrichi	Erysipelotrichales	Erysipelotrichaceae	Erysipelothrix	
OTU_1201	Bacteria	Firmicutes					
OTU_1155	Bacteria	Firmicutes					
OTU_605	Bacteria	Acidobacteria	Acidobacteria-6	CCU21			
OTU_762	Bacteria	Acidobacteria	Acidobacteria-6	iii1-15			
OTU_813	Bacteria	Acidobacteria	iii1-8	SJA-36			
OTU_489	Bacteria	Acidobacteria	Solibacteres	Solibacterales			
OTU_893	Bacteria	Acidobacteria	Solibacteres	Solibacterales			
OTU_467	Bacteria	Acidobacteria	Solibacteres	Solibacterales			
OTU_261	Bacteria	Armatimonadetes	[Fimbriimonadia]	[Fimbriimonadales]	[Fimbriimonadaceae]		
OTU_287	Bacteria	Chlorobi	BSV26	C20			
OTU_319	Bacteria	Chlorobi	Ignavibacteria	Ignavibacteriales	Ignavibacteriaceae		
OTU_317	Bacteria	Chlorobi	Ignavibacteria	Ignavibacteriales	Ignavibacteriaceae		
OTU_660	Bacteria	Chlorobi	Ignavibacteria	Ignavibacteriales	Ignavibacteriaceae		
OTU_1117	Bacteria	Chlorobi	OPB56				
OTU_782	Bacteria	Chlorobi	SJA-28				
OTU_157	Bacteria	Chlorobi	SJA-28				
OTU_201	Bacteria	Chloroflexi	Anaerolineae	A31	S47		
OTU_263	Bacteria	Chloroflexi	Anaerolineae	A31	S47		
OTU_589	Bacteria	Chloroflexi	Anaerolineae	S0208			
OTU_447	Bacteria	Chloroflexi	TK17	mle1-48			
OTU_686	Bacteria	Deferribacteres	Deferribacteres	Deferribacterales	Deferribacteraceae	Geovibrio	thiophilus
OTU_914	Bacteria	Fibrobacteres	Fibrobacteria	Fibrobacterales	Fibrobacteraceae	Fibrobacter	succinogenes
OTU_188	Bacteria	Nitrospirae	Nitrospira	Nitrospirales	Nitrospiraceae	Nitrospira	
OTU_490	Bacteria	Planctomycetes	[Brocadiae]	Brocadiales	Brocadiaceae	Candidatus Jettenia	
OTU_555	Bacteria	Planctomycetes	Phycisphaerae	Phycisphaerales			
OTU_1192	Bacteria	Spirochaetes	MVP-15	PL-11B10			
OTU_727	Bacteria	Synergistetes	Synergistia	Synergistales	Dethiosulfovibrionaceae	Aminobacterium	
OTU_308	Bacteria	Tenericutes	Mollicutes	Acholeplasmatales	Acholeplasmataceae		
OTU_913	Bacteria	Tenericutes	Mollicutes	Acholeplasmatales	Acholeplasmataceae	Acholeplasma	
OTU_153	Bacteria	Tenericutes	Mollicutes	Anaeroplasmatales	Anaeroplasmataceae	Anaeroplasma	
OTU_414	Bacteria	WPS-2					
OTU_1182	Bacteria	WPS-2					
OTU_1298	Bacteria						
OTU_682	Bacteria						
OTU_879	Bacteria						
OTU_1095	Bacteria						
OTU_463	Bacteria						
OTU_468	Bacteria						

OTU_413	Bacteria						
OTU_1232	Bacteria						
OTU_154	Bacteria						
OTU_900	Bacteria						
OTU_488	Bacteria						
OTU_837	Bacteria						

Shared

**OTUs**	**Kingdom**	**Phylum**	**Class**	**Order**	**Family**	**Genus**	**Specie**
OTU_16	Bacteria	Proteobacteria	Alphaproteobacteria	Rhizobiales	Phyllobacteriaceae		
OTU_18	Bacteria	Proteobacteria	Alphaproteobacteria	Rhodobacterales	Rhodobacteraceae	Anaerospora	
OTU_5	Bacteria	Proteobacteria	Alphaproteobacteria	Rhodobacterales	Rhodobacteraceae	Octadecabacter	
OTU_511	Bacteria	Proteobacteria	Alphaproteobacteria	Rhodobacterales	Rhodobacteraceae	Shimia	
OTU_249	Bacteria	Proteobacteria	Alphaproteobacteria	Rhodobacterales	Rhodobacteraceae		
OTU_2	Bacteria	Proteobacteria	Alphaproteobacteria	Rhodobacterales	Rhodobacteraceae		
OTU_11	Bacteria	Proteobacteria	Alphaproteobacteria	Rhodobacterales	Rhodobacteraceae		
OTU_47	Bacteria	Proteobacteria	Alphaproteobacteria	Rhodobacterales	Rhodobacteraceae		
OTU_78	Bacteria	Proteobacteria	Alphaproteobacteria	Rhodobacterales	Rhodobacteraceae		
OTU_769	Bacteria	Proteobacteria	Alphaproteobacteria	Rickettsiales	mitochondria		
OTU_182	Bacteria	Proteobacteria	Betaproteobacteria	Burkholderiales	Alcaligenaceae	Achromobacter	
OTU_9	Bacteria	Proteobacteria	Gammaproteobacteria	[Marinicellales]	[Marinicellaceae]		
OTU_20	Bacteria	Proteobacteria	Gammaproteobacteria	[Marinicellales]	[Marinicellaceae]		
OTU_3	Bacteria	Proteobacteria	Gammaproteobacteria	Alteromonadales	OM60		
OTU_7	Bacteria	Proteobacteria	Gammaproteobacteria	Alteromonadales	OM60		
OTU_32	Bacteria	Proteobacteria	Gammaproteobacteria	Alteromonadales			
OTU_8	Bacteria	Proteobacteria	Gammaproteobacteria	Enterobacteriales	Enterobacteriaceae	Escherichia	coli
OTU_1167	Bacteria	Proteobacteria	Gammaproteobacteria	Enterobacteriales	Enterobacteriaceae		
OTU_30	Bacteria	Proteobacteria	Gammaproteobacteria	Vibrionales	Pseudoalteromonadaceae	Pseudoalteromonas	
OTU_4	Bacteria	Proteobacteria	Gammaproteobacteria	Vibrionales	Vibrionaceae	Photobacterium	damselae
OTU_1	Bacteria	Proteobacteria	Gammaproteobacteria	Vibrionales	Vibrionaceae	Vibrio	
OTU_1100	Bacteria	Proteobacteria	Gammaproteobacteria	Vibrionales	Vibrionaceae	Vibrio	
OTU_23	Bacteria	Proteobacteria	Gammaproteobacteria	Vibrionales	Vibrionaceae	Vibrio	
OTU_22	Bacteria	Proteobacteria	Gammaproteobacteria	Vibrionales	Vibrionaceae	Vibrio	
OTU_61	Bacteria	Proteobacteria	Gammaproteobacteria				
OTU_170	Bacteria	Proteobacteria					
OTU_103	Bacteria	Proteobacteria					
OTU_44	Bacteria	Proteobacteria					
OTU_87	Bacteria	Bacteroidetes	Bacteroidia	Bacteroidales			
OTU_12	Bacteria	Bacteroidetes	Flavobacteriia	Flavobacteriales	Flavobacteriaceae	Tenacibaculum	
OTU_6	Bacteria	Bacteroidetes	Flavobacteriia	Flavobacteriales	Flavobacteriaceae		
OTU_35	Bacteria	Bacteroidetes	Flavobacteriia	Flavobacteriales	Flavobacteriaceae		
OTU_42	Bacteria	Bacteroidetes	Flavobacteriia	Flavobacteriales	Flavobacteriaceae		
OTU_49	Bacteria	Bacteroidetes	Flavobacteriia	Flavobacteriales	Flavobacteriaceae		

OTU_29	Bacteria	Bacteroidetes	Flavobacteriia	Flavobacteriales		
OTU_1062	Bacteria	Bacteroidetes	Flavobacteriia	Flavobacteriales		
OTU_148	Bacteria	Bacteroidetes	Flavobacteriia	Flavobacteriales		
OTU_33	Bacteria	Actinobacteria	Acidimicrobiia	Acidimicrobiales	C111	
OTU_10	Bacteria	Actinobacteria	Acidimicrobiia	Acidimicrobiales	koll13	
OTU_38	Bacteria	Actinobacteria	Actinobacteria	Actinomycetales	Cellulomonadaceae	Demequina
OTU_917	Bacteria	Actinobacteria	Actinobacteria	Actinomycetales	Cellulomonadaceae	Demequina
OTU_85	Bacteria	Firmicutes	Bacilli	Bacillales		
OTU_379	Bacteria	Firmicutes	Bacilli	Lactobacillales	Lactobacillaceae	Lactobacillus
OTU_936	Bacteria	Firmicutes	Clostridia	Clostridiales	Ruminococcaceae	Oscillospira
OTU_1018	Bacteria	Firmicutes	Clostridia	Clostridiales		
OTU_28	Bacteria	Verrucomicrobia	Verrucomicrobiae	Verrucomicrobiales	Verrucomicrobiaceae	Rubritalea
OTU_94	Bacteria	Verrucomicrobia	Verrucomicrobiae	Verrucomicrobiales	Verrucomicrobiaceae	
OTU_46	Bacteria	Verrucomicrobia	Verrucomicrobiae	Verrucomicrobiales	Verrucomicrobiaceae	
OTU_114	Bacteria					
OTU_111	Bacteria					
OTU_84	Bacteria					
OTU_25	Bacteria					
OTU_634	Unclassified					
OTU_98	Bacteria					
OTU_457	Bacteria					
OTU_88	Bacteria					
OTU_101	Bacteria					
OTU_192	Bacteria					
OTU_118	Bacteria					
OTU_145	Bacteria					
