# Supplementary material for: Exploring the Impact of the Biofloc Rearing System and an Oral WSSV Challenge on the Intestinal Bacteriome of Litopenaeus vannamei
Source: Microorganisms. 2018 Aug 8;6(3):83. doi: 10.3390/microorganisms6030083 (PMC6164277; doi:10.3390/microorganisms6030083)
Supplement: Supplementary file 1 [file microorganisms-06-00083-s001.zip › Table S1.pdf]

Table S1

**Total ammonia nitrogen** (mg. L^{-1})	**Nitrite** (N-NO_2 mg. L^{-1})	**Alkalinity** ($CaCO_3$ mg. L^{-1})	**Nitrate** (mg. L^{-1})	**pH**	**Total suspended solids** (mg. L^{-1})
0.82	0.72	176	62	8.41	427
