# Supplementary material for: Exploring the Impact of the Biofloc Rearing System and an Oral WSSV Challenge on the Intestinal Bacteriome of Litopenaeus vannamei
Source: Microorganisms. 2018 Aug 8;6(3):83. doi: 10.3390/microorganisms6030083 (PMC6164277; doi:10.3390/microorganisms6030083)
Supplement: Supplementary file 1 [file microorganisms-06-00083-s001.zip › Table S2.pdf]

Table S2

Sample	Raw PE	Combined	Qualified	Nochimera*	Q30	GC%
**BFT**	58,363	49,047	40,890	39,938	96.11	53.09
**CWS**	69,039	60,431	54,053	47,030	97.22	53.90
**BFT.W**	75,105	66,820	58,323	50,836	96.36	53.83
**CWS.W**	73,054	63,003	54,024	47,941	96.22	53.23
Total	275,561	239,301	207,290	185,745		
Mean±SD	68,890±6,458	59,825±6625	51,823±6,550	46,436±4,006	96,48±0,44	53,51±0,36

*Effective sequences used to downstream analysis.

Raw PE: paired-ends reads; **Q30**: paired-ends reads which passed through quality filter;

SD: standard deviation.
